# Multiparametric magnetic resonance imaging for detection of pathological changes in the central nervous system of a mouse model of multiple sclerosis in vivo

**DOI:** 10.1002/nbm.4964

**Published:** 2023-05-18

**Authors:** Abdullah A. Althobity, Nemat Khan, Cheyenne J. Sandrock, Trent M. Woodruff, Gary J. Cowin, Ian M. Brereton, Nyoman D. Kurniawan

**Affiliations:** ^1^ Centre for Advanced Imaging The University of Queensland Brisbane Australia; ^2^ Al Azhar Hospital Riyadh Saudi Arabia; ^3^ Society of Artificial Intelligence in Healthcare Riyadh Saudi Arabia; ^4^ Department of Radiological Sciences and Medical Imaging, College of Applied Medical Sciences Majmaah University Majmaah Saudi Arabia; ^5^ Faculty of Medicine, School of Biomedical Sciences The University of Queensland Brisbane Australia; ^6^ Queensland Brain Institute The University of Queensland Brisbane Australia; ^7^ NCRIS Australian National Imaging Facility The University of Queensland Brisbane Australia

**Keywords:** diffusion‐weighted magnetic resonance imaging, experimental autoimmune encephalomyelitis, mouse spinal cord, multiple sclerosis

## Abstract

Multiple sclerosis (MS) is an autoimmune disease involving demyelination and axonal damage in the central nervous system (CNS). In this study, we investigated pathological changes in the lumbar spinal cord of C57BL/6 mice induced with progressive experimental autoimmune encephalomyelitis (EAE) disease using 9.4‐T magnetic resonance imaging (MRI). Multiparametric MRI measurements including MR spectroscopy, diffusion tensor imaging (DTI) and volumetric analyses were applied to detect metabolic changes in the CNS of EAE mice. Compared with healthy mice, EAE mice showed a significant reduction in *N*‐acetyl aspartate and increases in choline, glycine, taurine and lactate. DTI revealed a significant reduction in fractional anisotropy and axial diffusivity and an increase in radial diffusivity in the lumbar spinal cord white matter (WM), while in the grey matter (GM), fractional anisotropy increased. High‐resolution structural imaging also revealed lumbar spinal cord WM hypertrophy and GM atrophy. Importantly, these MRI changes were strongly correlated with EAE disease scoring and pathological changes in the lumbar (L2–L6), particularly WM demyelination lesions and aggregation of immune cells (microglia/macrophages and astrocytes) in this region. This study identified changes in MRI biomarker signatures that can be useful for evaluating the efficacy of novel drugs using EAE models in vivo.

## Graphical abstract

We used multimodal parametric MRI using cryoprobe at 9.4 T to study chronic EAE pathology in the mouse lumber spinal cord. We reported new MRI biomarkers including increased taurine and glycine and increased FA in the GM. We also observed increases in choline and RD which indicate demyelination, reductions in *N*‐acetyl aspartate and AD which indicate axonal damage, and atrophy in GM and hypertrophy in WM. These lumbar MRI biomarkers have a significant association with EAE disease severity.

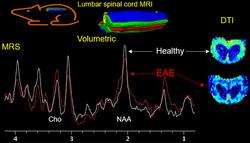

**Abbreviations**
BSA: bovine serum albumin
CFA: complete Freund's adjuvant
CNS: central nervous system
CSDE: chemical shift displacement error
DAPI: 4′,6‐diamidino‐2‐phenylindole
DF: dorsal funiculus
DL: deep learning
EAE: experimental autoimmune encephalomyelitis
FM: FluoroMyelin
FWHM: full width at half maximum
GFAP: glial fibrillary acidic protein
GPC: glycerophosphocholine
IHC: immunohistochemistry
L1‐L6: lumbar spinal cord segments from 1 to 6
LF: lateral funiculus
MOG: myelin oligodendrocyte glycoprotein
MS: multiple sclerosis
NAWM: normal appearing white matter
PBS: phosphate‐buffered solution
SCT: Spinal Cord Toolbox
T13: thoracic level 13
USR: ultra‐shielded and refrigerated
VF: ventral funiculus
WC: whole cord
WT: wild type

## 1 INTRODUCTION

Multiple sclerosis (MS) is a chronic autoimmune disorder of the central nervous system (CNS) that often leads to sensory, motor and cognitive deficits.^1^ MS occurs when the immune system mistakenly recognises components of myelin, oligodendrocytes or neurons as foreign bodies, and this autoimmunity reaction leads to demyelination and neurodegeneration.^2^ Mouse models of experimental autoimmune encephalomyelitis (EAE) have been widely used to characterise pathophysiological aspects of MS disease progression that can exhibit monophasic progressive or relapsing‐remitting disease based on the induction method.^3^, ^4^

Magnetic resonance imaging (MRI) has been used extensively for clinical diagnosis of MS in the brain (see recent consensus on the use of MRI in MS^5^), but less data are available for the spinal cord. Multimodal parametric MRI using T_1_‐weighted,^6^ T_2_‐weighted^7^ and proton density (PD)^8^ imaging, as well as magnetic resonance (MR) spectroscopy (MRS),^9^, ^10^ have been used in separate studies to reveal MS pathological changes in the human spinal cord.

MS disease in human brain resulted in metabolic changes of *N*‐acetyl aspartate (NAA),^11^, ^12^, ^13^ choline (Cho)^11^, ^14^ and myo‐inositol (m‐Ins),^13^, ^14^ with similar results reported for the human spinal cord.^9^, ^10^, ^15^, ^16^, ^17^, ^18^, ^19^ The basal metabolite levels in the brain and spinal cord, however, are different. In healthy individuals, NAA was found to be lower in spinal cord compared with the brainstem and in the cortex, whereas creatine (Cr) was lower in the spinal cord compared with the cerebellum.^20^ NAA, Cho and m‐Ins were also reported to be lower in the spinal cord compared with the brainstem.^21^

The characteristics of EAE mouse models have been studied using various modalities of MRI.^22^, ^23^, ^24^ However, no MRS study characterising metabolite changes in the EAE lumbar spinal cord has been reported to date, and the use of MRS to study spinal cord metabolites in rodent models has been limited.^25^, ^26^ More recently, an MRS study at 9.4 T compared lumbar spinal cord spectra from healthy mice and rats.^27^ The use of a cryoprobe coil permitted the acquisition of signal from a much smaller (four times) voxel size in the mice and achieved better spectral resolution compared with that obtained from rats.^27^

Diffusion tensor imaging (DTI) studies of EAE mouse lumbar spinal cord at 4.7 T have reported anisotropy changes in the white matter (WM) structures.^28^ However, DTI changes in the spinal cord grey matter have not been reported. In GM, the fractional anisotropy (FA) value is generally low (~0.2) and close to noise, which may make it difficult to measure changes accurately because of low signal‐to‐noise ratio (SNR).

Recent studies of the brain in EAE mice reported that the brain volumes increased during peak EAE disease followed by atrophy at the chronic phase.^22^ Atrophy was detected in the brain GM but not in the WM.^23^, ^29^ It is expected that EAE‐induced demyelination and axonal loss will eventually lead to atrophy, but such volumetric changes have not been reported in the spinal cord of EAE mice. Because of its small structures, high‐resolution anatomical imaging is needed to accurately measure atrophy in the spinal cord.

We hypothesise that a multiparametric MR approach at 9.4 T will identify new markers of EAE pathology that correlate with the animal scores. The current study focused on the lumbar spinal cord as the EAE pathology has been reported to mainly affect the lumbar region.^30^, ^31^

A cryoprobe was employed to optimise SNR so that high‐resolution imaging could be used to detect volumetric changes in the WM, that MRS could be achieved in a feasible acquisition time and that changes in GM could be detected using DTI. These capabilities are important, as MS affects both the GM and the WM.^1^, ^32^ These methods were utilised to quantitate differences between healthy and EAE mice. MRI findings were validated using immunohistochemistry (IHC) to assess their sensitivity to EAE pathology.

## 2 MATERIALS AND METHODS

### 2.1 Animal model and preparation

All the mice were housed and handled according to the Queensland Animal Care and Protection Act 2001 and the current NHMRC Australian Code of Practice for the Care and Use for Scientific purposes, and the experiments were performed following approval from the University of Queensland animal ethics committee.

Wild‐type female C57BL6/J mice (*n* = 36; age = ~12–14 weeks) acquired from the Animal Resources Centre (Perth, Australia) were induced with EAE. Age‐matched C57BL6/J mice receiving no treatments were used as a naïve control group. During the current study, we used 15 mice for the healthy group and 21 mice for EAE induction. For EAE disease induction, each animal received a subcutaneous injection of myelin oligodendrocyte glycoproteins (MOG_35–55_) (100 μg) emulsified in complete Freund's adjuvant (CFA) (4 mg/mL). The MOG_35–55_/CFA emulsion was injected as 100 μL (equally divided over two flanks), followed by intraperitoneal injection of 100 μL (200 ng) of pertussis toxin.^33^ A five‐point scoring system^34^ (Table S1) was used to assess the animals daily. EAE model induction and scoring were carried out by three independent assessors (A.A., N.K. and C.S.). Animals were scanned by MRI at 21 ± 3 days postimmunisation, a peak EAE disease timepoint whereby animals exhibited scores of 1.5–3 (representing partial or complete hindlimb paralysis, respectively).

### 2.2 MR protocols

All MR data were acquired using a Bruker Biospec 9.4‐T 30‐cm Ultra Shielded and Refrigerated animal MRI system equipped with an actively shielded BGA12s gradient set (maximum gradient strength 660 mT/m with 3440 T/m/s maximum slew rate). The scanner was equipped with a mouse head quadrature Tx/Rx cryoprobe surface coil for 6‐cm gradients and operated under Paravision 6.0.1 software (Bruker BioSpin, Karlsruhe, Germany).

Ancillary instruments consisted of an anaesthetic system (MEDIQUIP), an animal physiology monitoring system (SA Instruments, Inc, model:110100 ERT Rev A) with two sensors for breathing and temperature, and warm water circulation for the animal bed and cryoprobe coil (Thermo Scientific), which were all configured and equilibrated before bringing the animals to the scanner room. Foam spacers were placed in the cryoprobe bed to aid animal positioning and to ensure the lumbar cord was as straight as possible. Respiration and temperature sensors were securely taped under the mouse abdomen. Prior to positioning in the cryoprobe, the mouse was placed in an anaesthesia chamber filled with a 3% isoflurane and medical oxygen air mixture at a flow rate of 2 L/min. Upon anaesthesia, the mouse was placed on the cryoprobe bed, positioned prone and headfirst, with a tooth hook to immobilise the head. An isoflurane/air mixture of 1.1%–1.8%, with the balance being the medical oxygen, was delivered through a head cone at a flow rate of 0.7 L/min. The mouse position was checked by locating the bed in a mock cryoprobe outside the scanner to ensure that the lumbar cord was at the coil isocentre and that the mouse could be safely inserted into the cryoprobe. During imaging, animal respiration was maintained at 40–60 beats per minute and body temperature at 34°C. Mouse positioning for lumbar spinal cord imaging using a cryoprobe is shown in Figure S1.

#### 2.2.1 MRS

Shimming was initiated using a 10‐mm first‐order shim radius located at the magnet centre, coinciding with the spinal lumbar region. A three‐axis localisation scan was first used to locate the lumbar spinal cord region for planning the MRS and MRI protocols. A fast low angle shot (FLASH) sequence was used for PD weighting with parameters: TR = 500 ms, TE = 2.5 ms and flip angle = 45°, NEX = 2, slice thickness = 0.5 mm and FOV = 10 × 10 mm. The scan time was 3 min with respiratory gating. The RF power was manually adjusted to obtain homogeneous images of the lumbar section.

**FIGURE 1:**
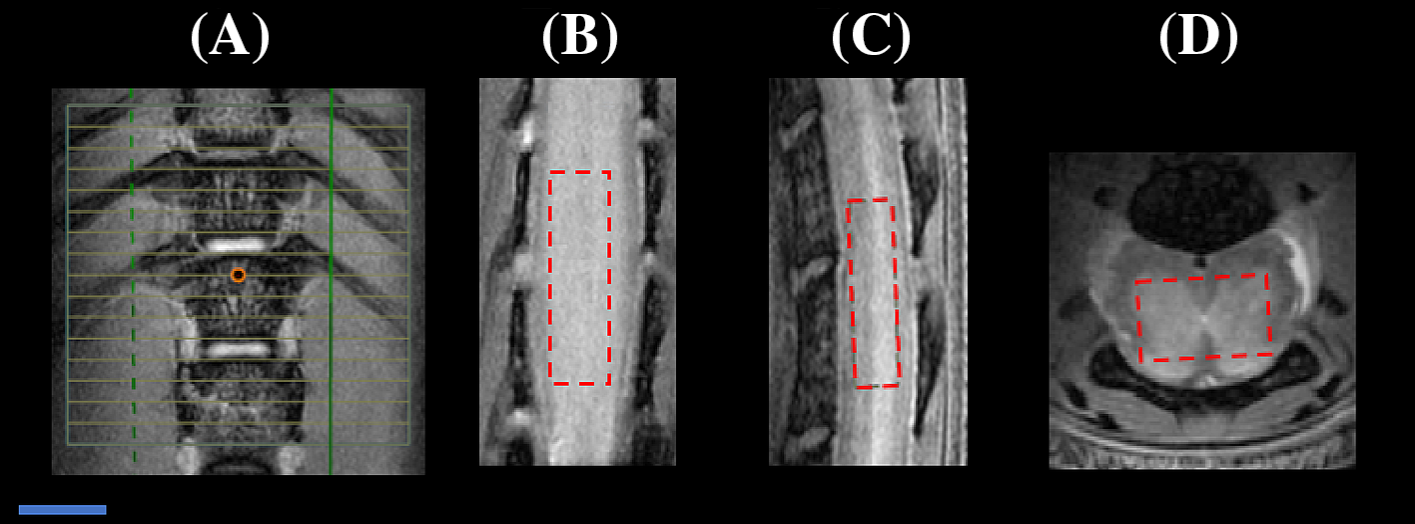
Planning imaging for PD imaging, DTI and MRS on healthy mouse. (A) The centre of the slice package (slice #8, marked with an orange dot) was placed on the last thoracic vertebral body (T13) for planning PD imaging and DTI. A high‐resolution PD image was used for localisation of the MRS voxel, primarily within the lumbar spinal cord GM. The MRS voxel dimensions were 2.8 × 1.6 × 0.9 mm (Z, X and Y directions) as shown on (B) Coronal, (C) Sagittal, and (D) Axial views. The position of the MRS voxel matches the nine slices used for PD imaging and DTI analysis. Scale bar is 1 mm. DTI, diffusion tensor imaging; GM, grey matter; MRS, magnetic resonance spectroscopy; PD, proton density.

MRS was acquired using a Point Resolved Spectroscopy (PRESS) sequence. The spectroscopy voxel was placed in the GM guided by high‐resolution axial/sagittal/coronal images (Figure 1). Most of the spectroscopy signal contained signal originating from the GM, with some signal from the WM. The signals from the WM are from WM edges that were unavoidable due to the butterfly shape of the GM (Figure 1D). To ensure reproducibility, the MRS voxel was always placed at the same location, as shown in Figure 1B–D. The centre of the MRS voxel was placed at the last thoracic vertebral body (T13), which is the largest and least curved section of the spine. At this location, the voxel position is already constrained, close to the spine bony structure. Distal and rostral to this area, the spine is curved and narrowing, not permitting voxel placement in this area.

A local first‐order shimming protocol was applied to the voxel, followed by a local frequency adjustment to place the water signal on resonance. This localised shim protocol was used to reduce the water signal full width at half maximum (FWHM) to 23 ± 3 Hz. A misplacement of the voxel can be identified with bad shimming, resulting in large linewidth of the water peak during the acquisition of the spectrum without water suppression. Bruker BioSpin's TOPSPIN (version 3.1 PV) was used to analyse the spectral quality; eight spectra with FWHM of more than 28 Hz were excluded from analysis.

To minimise the chemical shift displacement error (CSDE),^35^ the bandwidth (BW) of the PRESS localisation RF pulses was set at 8400 Hz, the highest feasible value for the scanner. The approximate NAA to water CSDE was relatively low (12%). Variable Pulse Power and Optimized Relaxation Delays (VAPOR) was chosen for water suppression as it produced better suppression compared with Chemical Shift Selective Suppression (CHESS) (data not shown). The final MRS parameters were TR/TE = 2500/14 ms, voxel dimensions and size = 1.6 × 0.9 × 2.8 mm^3^ (4.03 μL), RF pulse BW = 8400 Hz, NEX = 128 and acquisition time = 8 min with respiratory gating.

In the initial experiments, MRS was performed using voxels placed on both the hippocampus and the lumbar spinal cord to determine whether there were any metabolite concentration differences between the two CNS organs. Four healthy mice were scanned using the same MRS protocol at TE = 14 and 30 ms to compare the glycine (Gly) and m‐Ins peaks in vivo with equivalent phantom data. At TE = 30 ms, Gly was detectable in the lumbar spinal cord, but was significantly reduced compared with spectra obtained at TE = 14 ms (Figure S2). At TE = 30 ms, the m‐Ins region exhibited low signal with extremely high SD (not detectable in all four samples), as predicted from scalar coupling evolution considerations. As TE = 30 ms could not be used to measure both Gly and m‐Ins reliably, subsequent MRS on the cohort animals used only TE = 14 ms to minimise the total experiment time.

#### 2.2.2 High‐resolution axial PD and DTI

The upper edge of the last thoracic vertebral body (T13) joint with the ribs was used as an anatomical landmark for all MRI slice positioning (Figure 1), consistent with the localisation within the lumbar spinal cord.^36^ For PD‐weighted imaging, the following parameters were used: TR/TE = 500/2.85 ms, flip angle 45°, resolution 40 × 40 μm, 16 slices with slice thickness = 0.5 mm, FOV = 10 × 10 mm, NEX = 4, BW = 60 KHz and acquisition time = 12 min with respiratory gating. DTI data were acquired with matching position and geometry as the PD‐weighted image using a two‐dimensional (2D) diffusion‐weighted spin‐echo sequence with nine orthogonal diffusion directions with b = 1500 s/mm^2^ and two b = 0 images, with TR/TE = 800/18 ms, resolution 78 × 78 μm, NEX = 1, BW = 45 KHz and acquisition time = 23 min with respiratory gating.

### 2.3 MRS and MRI processing

#### 2.3.1 MRS

Linear combination modelling (LCModel) version 6.3 (s‐provencher.com/lcmodel.shtml) was used for spectral analysis to determine metabolite concentrations. At the beginning of the sequence, unsuppressed water signal was taken from the same voxel and used as a reference for water scaling and metabolite concentration quantification. Cr was chosen as an internal reference because our data did not show any significant difference of the absolute Cr concentrations between healthy and diseased mice. Cr has been found to be substantially invariant in MS compared with healthy volunteers.^11^ The metabolite basis set at TE = 14 ms was obtained from the LCModel source^37^ to match our PRESS parameters. This basis set was used to fit the acquired spectra using a linear combination, and rejection criteria for fitting a particular metabolite used a threshold standard deviation of more than 15%.

NAA is quantified as a total of *N*‐acetylaspartylglutamate (NAAG) + NAA, and Cho as a total of glycerophosphocholine (GPC) + phosphocholine (PCh), as these total signal measures were suggested to be more accurate than when attempting to separate the overlapped individual metabolites.^37^ The LCModel program was configured by adding Gly to the basis set. ‘NSIMUL = 14’ was added in the script to include metabolites of interest: phosphocreatine (PCr), gamma‐aminobutyric acid (GABA), glutamate (Glu), glutamine (Gln), glutathione (GSH), taurine (Tau), m‐Ins, lactate (Lac), NAA, Gly, GPC + PCh, NAA + NAAG, m‐Ins + Gly, Cr + PCr and Glu + Gln. The spectra were analysed from 0.2 to 4.2 ppm instead of the default settings (0.0–4.00 ppm) to include m‐Ins at 4.06. Nuclear magnetic resonance spectra were generated through simulations to understand the components and deconvolution of all metabolite peaks in a spectrum; these assessments are shown in Figure S3.

#### 2.3.2 MR image analysis

FA, axial diffusivity (AD), mean diffusivity (MD) and radial diffusivity (RD) maps were calculated using FMRIB Software Library (FSL)^38^ DTIFIT. Motion and eddy current correction were not performed, as trials using FSL registration tools resulted in image misregistration. The effects of motion artefacts have been minimised during acquisition using respiratory gating, and furthermore, slices with artefacts were excluded from analysis. Diffusion‐weighted images were acquired using a moderate b‐value (b = 1500 s/mm^2^) and using a standard spin‐echo readout sequence (i.e., not using echo planar imaging). Using this setting, we did not detect eddy current distortion, which otherwise can be observed as a halo in the DTI parametric maps.

The Spinal Cord Toolbox (SCT) deep learning (DL) model for the spinal cord WM/GM segmentation^39^ was retrained using manually segmented mouse spinal cord data (*n* = 13) by ITK‐SNAP^40^ (version 3.8.0). The retraining of SCT was required to enable it to segment the GM and WM structures of the mouse lumbar spinal cord (assisted by Prof. Julien Cohen‐Adad and Charley Gros, Polytechnique Montreal, Canada). Subsequently, region of interest (ROI) segmentation of the spinal cord WM and GM was performed on the high‐resolution PD‐weighted images using this retrained SCT module. In five samples, manual corrections were made to remove segmentation errors. WM was further segmented into three ROIs: the ventral funiculus (VF), lateral funiculus (LF) and dorsal funiculus (DF). High‐resolution PD axial imaging was used to measure the areas of WM and GM. Segmented ROIs were registered into DTI maps by affine registration of the PD image onto the DWI b = 0 image using FSL FLIRT, and DTI parameters were measured using ITK‐SNAP. Sixteen slices from all groups with observed geometric distortion or motion artefacts were excluded from analysis. DTI parameters were measured within the two main regions of the spinal cord (whole GM and WM), as well as three subregions of the WM (VF, LF and DF). To measure the volumes of normal‐appearing WM (NAWM), a FA threshold of more than 0.5 was used. Area measurements were made using nine slices that covered the lumbar enlargement. The results of SCT DL segmentation and ROI registrations are shown in Figures S6 and S7.

### 2.4 Immunohistochemistry

#### 2.4.1 Tissue collection and sectioning

Following MRI, the mice were euthanised using an overdose of pentobarbitone (~25 μL of 325.73 g/L stock solution). The mice were immediately perfused using 25 mL of phosphate‐buffered solution (PBS), followed by 25 mL of 4% paraformaldehyde (PFA) in 1 × PBS, pH 7.4. The mouse lumbar spinal cord section (L3–L5) was excised and postfixed in 4% PFA in PBS overnight, followed by washing and storage in PBS/Na‐azide (0.1%) solution. This section was imbedded in agarose 4% in distilled water and cut using a vibratome (Leica vibratome VT 1000 S, at the Centre for Advanced Imaging) at 30‐μm slice thickness. This method produced a complete series of axial slices from L3 to L5, covering the same area used for PD/DTI/MRS quantification (Figure 1). Histological staining was performed using slices taken randomly from within this lumbar section.

#### 2.4.2 Immunofluorescence staining

Sections were washed twice in 0.1% PBST (PBS + 0.1% Tween 20 detergent) for 10 min to remove agarose surrounding the spinal cord. This was followed by incubation with 5% bovine serum albumin (BSA) (Sigma‐Aldrich; catalogue number: A7906) and 0.1% Triton X detergent to prevent nonspecific antibody binding (by decreasing background staining) and permeabilise the tissue prior to adding antibodies.^41^ Primary antibodies prepared in 1% BSA at appropriate dilutions (as described below) were used for overnight incubation at 4°C. FluoroMyelin (FM) (Invitrogen; catalogue number: F34651) was used at 1:300 dilution for staining the myelin sheath and to investigate the extent of demyelination in all mice groups. Rabbit anti‐ionised calcium binding adaptor molecule 1 (Iba‐1) (Wako; catalogue number: 019‐19741) was used at 1:500 dilution to detect the activation of microglia/macrophages in lumbar spinal cord sections. Rabbit anti‐glial fibrillary acidic protein (GFAP) (Cell Signaling Technology; catalogue number: 12389) was used at 1:1000 dilution to detect astrocyte activation. Antirabbit Alexa Fluor 647 (Invitrogen; catalogue number: A‐21245) (1:800) diluted in 0.1% PBST was used to detect Iba‐1 and GFAP antibodies. Finally, 4′,6‐diamidino‐2‐phenylindole dihydrochloride (DAPI, blue fluorescence, Sigma‐Aldrich catalogue number: D9542) 1:5000 in PBS was used to detect nuclei in all sections.

#### 2.4.3 Microscopic imaging

Slides were imaged using SP8 laser point scanning confocal microscopy (Leica DM18 SP8). Slide scanning was performed using 20 × objective magnification, a 2D (XY) bidirectional scan, a scan speed of 600 Hz, and the line average was 4. Image dimensions were 775 × 775 μm^2^, with pixel size 0.758 × 0.758 μm^2^; 63 images were stitched together to obtain a final tiled image. Immunofluorescence images were processed using Leica Application Suite software (version 3.5.1.) and measured qualitatively.

### 2.5 Statistical analysis

All statistical analyses were carried out in Prism 9.3.1 (GraphPad Software, La Jolla, CA, USA). Comparisons of MRI and EAE score results between all groups were performed using the *t*‐test. All *p* values below 0.05 were considered significant, and the significance level is indicated as: ns = *p* > 0.05; * = *p* ≤ 0.05; ** = *p* ≤ 0.01; *** = *p* ≤ 0.001; **** = *p* < 0.0001. The normality tests were carried out using QQ plot, and all data were normally distributed. Pearson's correlation coefficient was used to measure the statistical relationship between MRS, DTI and EAE scores. Values of r of 0–0.19 were regarded as very weak, 0.2–0.39 as weak, 0.40–0.59 as moderate, 0.6–0.79 as strong and 0.8–1 as very strong correlation.^42^

## 3 RESULTS

The EAE disease course started at about Day 6 after the induction and reached the mean peak score (2.5 ± 0.5) at Day 20 ± 3, corresponding to the MRI scan period. Healthy mice did not develop any symptoms. Daily EAE disease scoring progression is shown in Figure S8.

### 3.1 MRS

**FIGURE 2:**
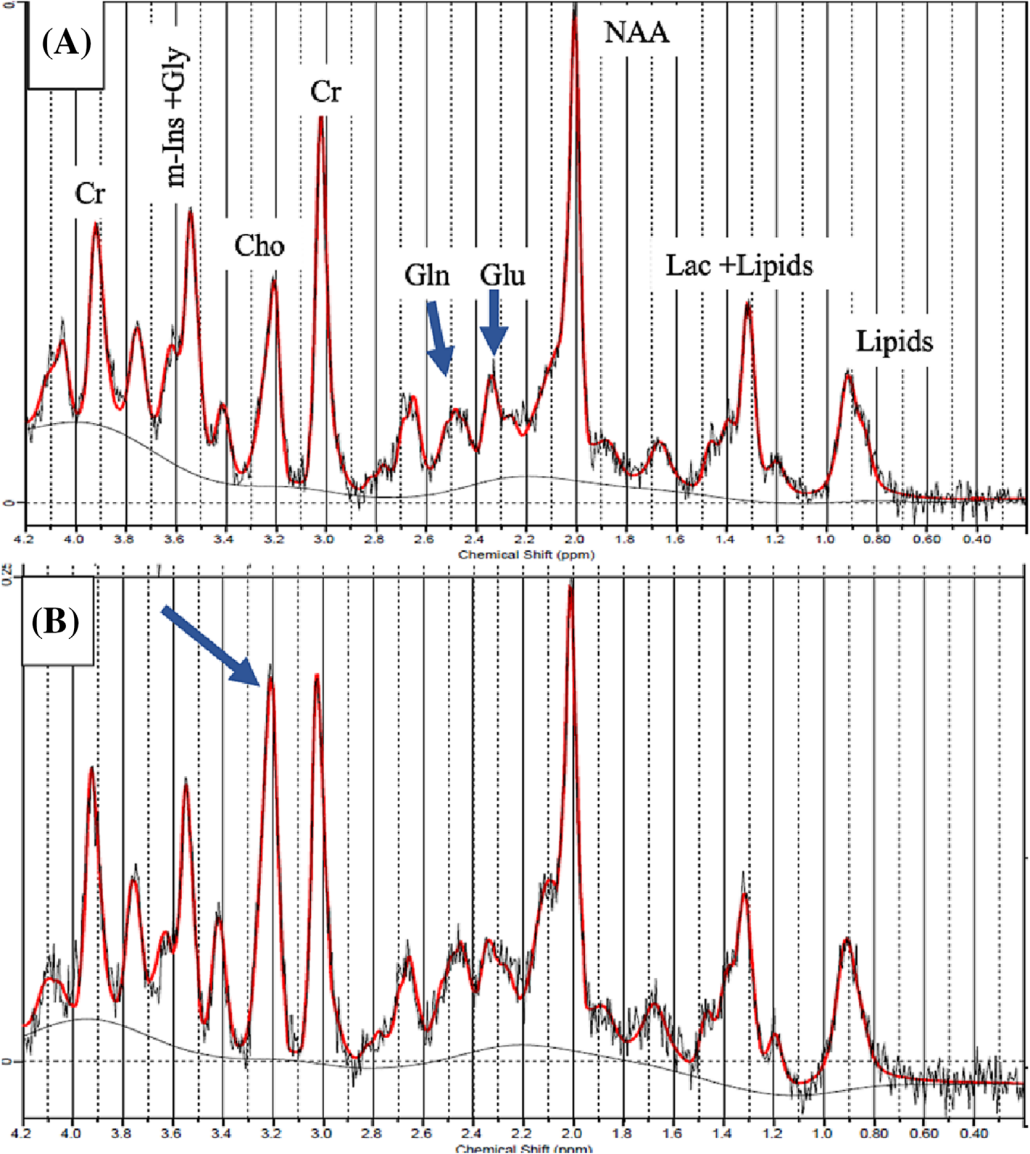
MRS spectra for (A) Healthy, and (B) EAE mice. The arrow in (B) indicates a significant increase of Cho in the EAE mice. Cho, choline; Cr, creatine; EAE, experimental autoimmune encephalomyelitis; Gln, glutamine; Glu, glutamate; Gly, glycine; Lac, lactate; m‐Ins, myo‐inositol; MRS, magnetic resonance spectroscopy; NAA, *N*‐acetyl aspartate.

Representative MR spectra quality and metabolite peak assignment for healthy and EAE mice are shown in Figure 2A,B.

#### 3.1.1 Quantification of Gly and m‐Ins in the spinal cord

Initial LCModel analysis of spinal cord data resulted in poor fitting for m‐Ins. Adding Gly to the spectral basis set significantly improved LCModel fitting, as shown in Figure S4. It is usually observed that the m‐Ins concentration is higher than Gly in mouse brain.^43^ To confirm these apparent conflicting observations, we acquired spectra from the hippocampus region for healthy mice using the same protocol as described for the spinal cord measurements. For spectra acquired in the mouse brain, addition of Gly to the LCModel basis set did not improve the quality of fit but resulted in a high SD. This contrasts with spectra acquired in the lumbar area, where Gly was reliably measured. In contrast to Gly, m‐Ins signal was detected in all brain spectra. MR spectra of the brain fitted with and without Gly in the LCModel basis set are shown in Figure S5.

**FIGURE 3:**
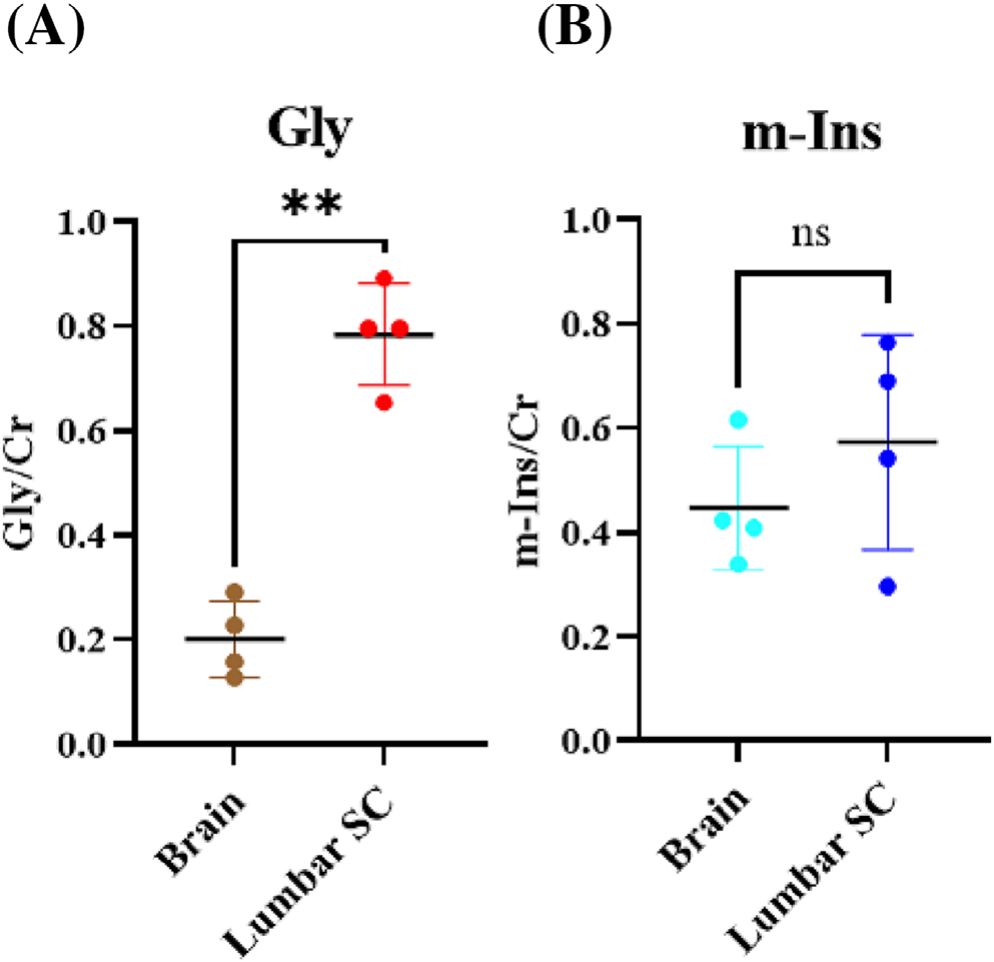
Comparison between Gly/Cr and m‐Ins/Cr in the brain and the lumbar spinal cord (SC). (A) Gly/Cr was significantly higher in the lumbar SC compared with the brain. (B) Similar m‐Ins/Cr levels were measured in the brain and lumbar regions. Cr, creatine; Gly, glycine; m‐Ins, myo‐inositol. **p < 0.01.

Analysis of Gly/Cr and m‐Ins/Cr in the brain and the lumbar spinal cord of healthy mice showed that (i) Gly/Cr was higher in the mouse lumbar spinal cord (0.79 ± 0.09) than in the brain (0.2 ± 0.07, *p* = 0.001), and (ii) the mouse brain contained higher m‐Ins/Cr (0.45 ± 0.12) than Gly/Cr (0.2 ± 0.07, *p* = 0.013) (Figure 3). LCModel fitting of the MR spectra of mouse brain and spinal cord is shown in Figure S9.

#### 3.1.2 Metabolite changes in EAE

**FIGURE 4:**
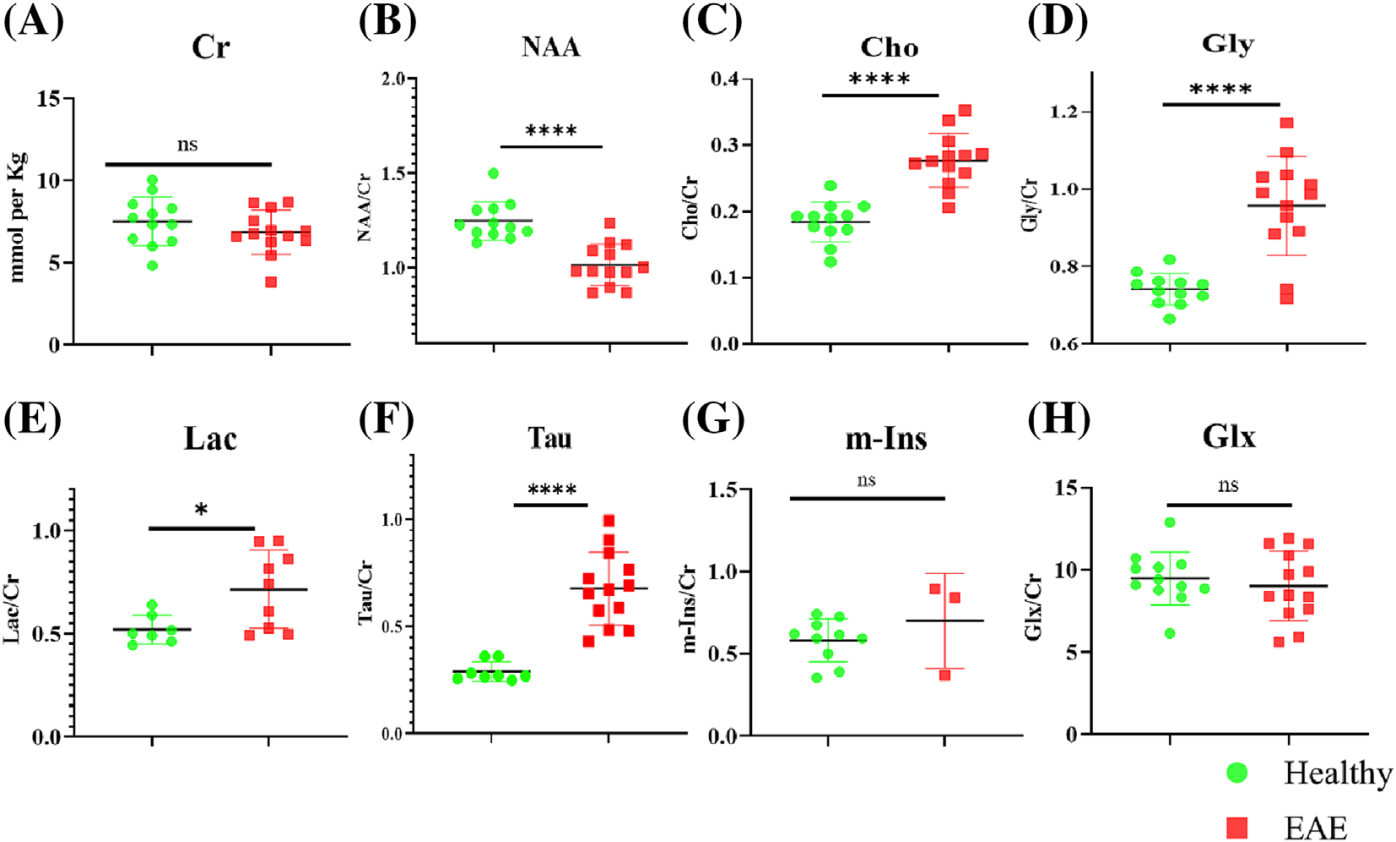
Metabolite measurements in the lumbar spinal cord of healthy and EAE mice. (A) Cr concentrations were measured by LCModel and did not show any significant change. (B–F) The ratios of NAA/Cr, Cho/Cr, Gly/Cr, Tau/Cr and Lac/Cr showed significant changes between healthy and EAE animals. A significant reduction in NAA/Cr and a significant increase in Cho/Cr, Gly/Cr, Lac/Cr and Tau/Cr were observed in EAE mice compared with healthy mice. (G) In m‐Ins, only three samples from EAE mice were recorded and hence no significant change was reported. (H) Glu + Gln were summed as Glx and show no change. Error bars indicate group mean ± SD. Cho, choline; Cr, creatine; EAE, experimental autoimmune encephalomyelitis; Gln, glutamine; Glu, glutamate; Gly, glycine; Lac, lactate; m‐Ins, myo‐inositol; NAA, *N*‐acetyl aspartate; Tau, taurine. *p < 0.05, ****p < 0.0001.

Cr concentration relative to water did not appear to vary between the EAE and the healthy animals (Figure 4A) and thus was used to normalise the concentrations of the other metabolites. NAA/Cr ratio was significantly lower in the EAE (1.0 ± 0.1) compared with healthy mice (1.25 ± 0.1, *p* < 0.0001). This decrease was concomitant with a highly significant increase in the Cho/Cr ratio for the EAE (0.28 ± 0.04) compared with the healthy mice (0.18 ± 0.03, *p* < 0.0001). Interestingly, Gly/Cr was significantly higher in the EAE (0.96 ± 0.13) compared with healthy mice (0.74 ± 0.04, *p* < 0.0001).

EAE mice exhibited higher Lac/Cr and Tau/Cr (0.72 ± 0.19 and 0.68 ± 0.17, respectively) compared with healthy mice (0.52 ± 0.07, *p* < 0.05 and 0.29 ± 0.04, *p* < 0.0001, for Lac/Cr and Tau/Cr, respectively). The relative concentrations of Glu and Gln were difficult to quantify individually and were analysed as Glx (Glu + Gln), but they did not show any change between the groups. MRS findings are summarised in Figure 4. Measurements of the metabolites relative to water (not relative to Cr) can be found in the supporting information (Supplementary materials 10, Figure S16).

### 3.2 DTI

**FIGURE 5:**
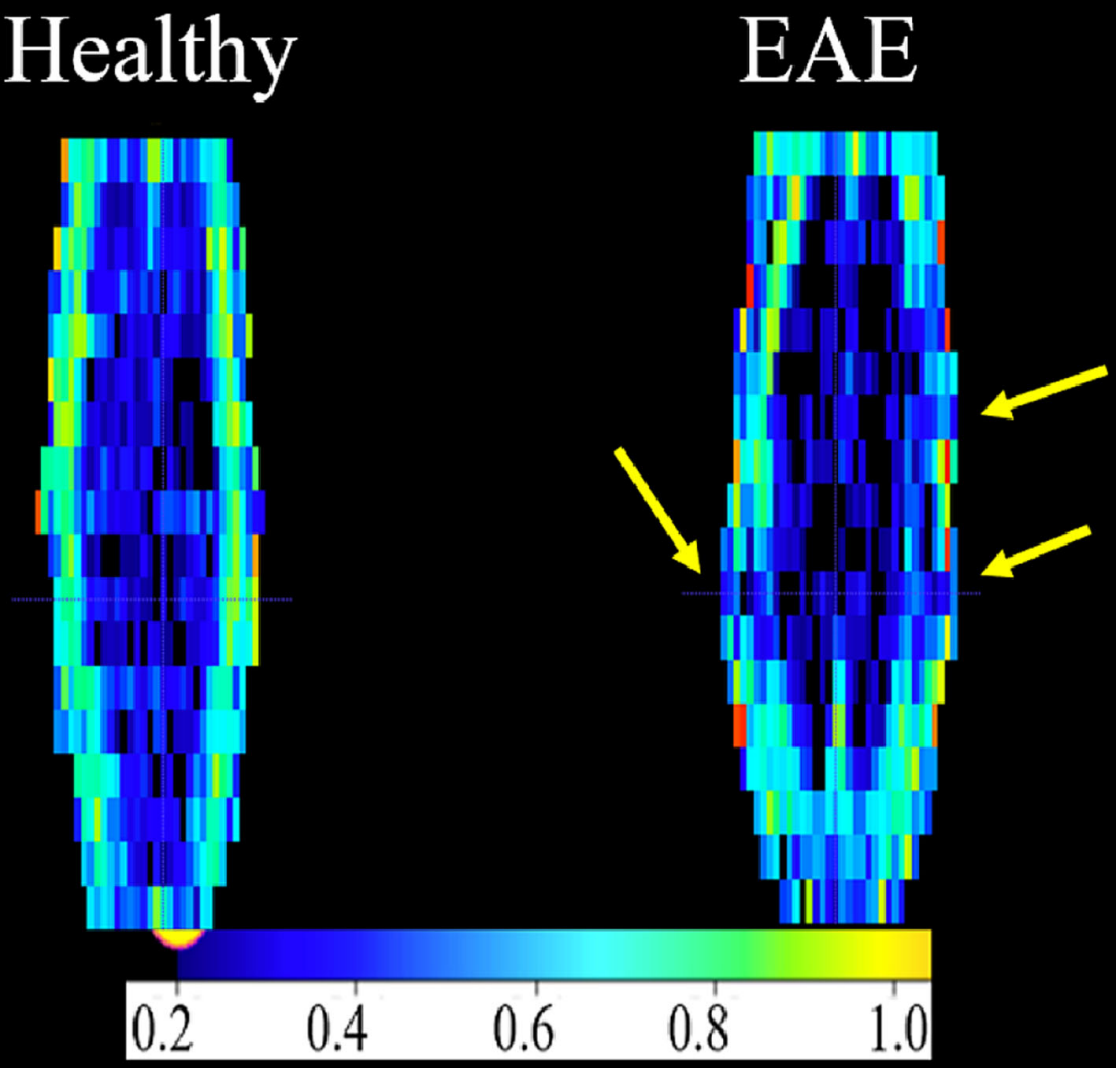
Coronal FA maps of the lumbar spine. Images were reconstructed from axial slices. WM can be seen in teal colour and GM in deep blue colour. Lesion areas can be seen as a localised decrease in FA (loss of hyperintense teal colour), as indicated by yellow arrows. EAE, experimental autoimmune encephalomyelitis; FA, fractional anisotropy; GM, grey matter; WM, white matter.

DTI maps, particularly of the FA, can visualise the location of EAE lesions along the spinal cord (Figure 5). The lesions appeared mainly on L3–L4, which was covered by the length of the MRS voxel (Figure 1).

**FIGURE 6:**
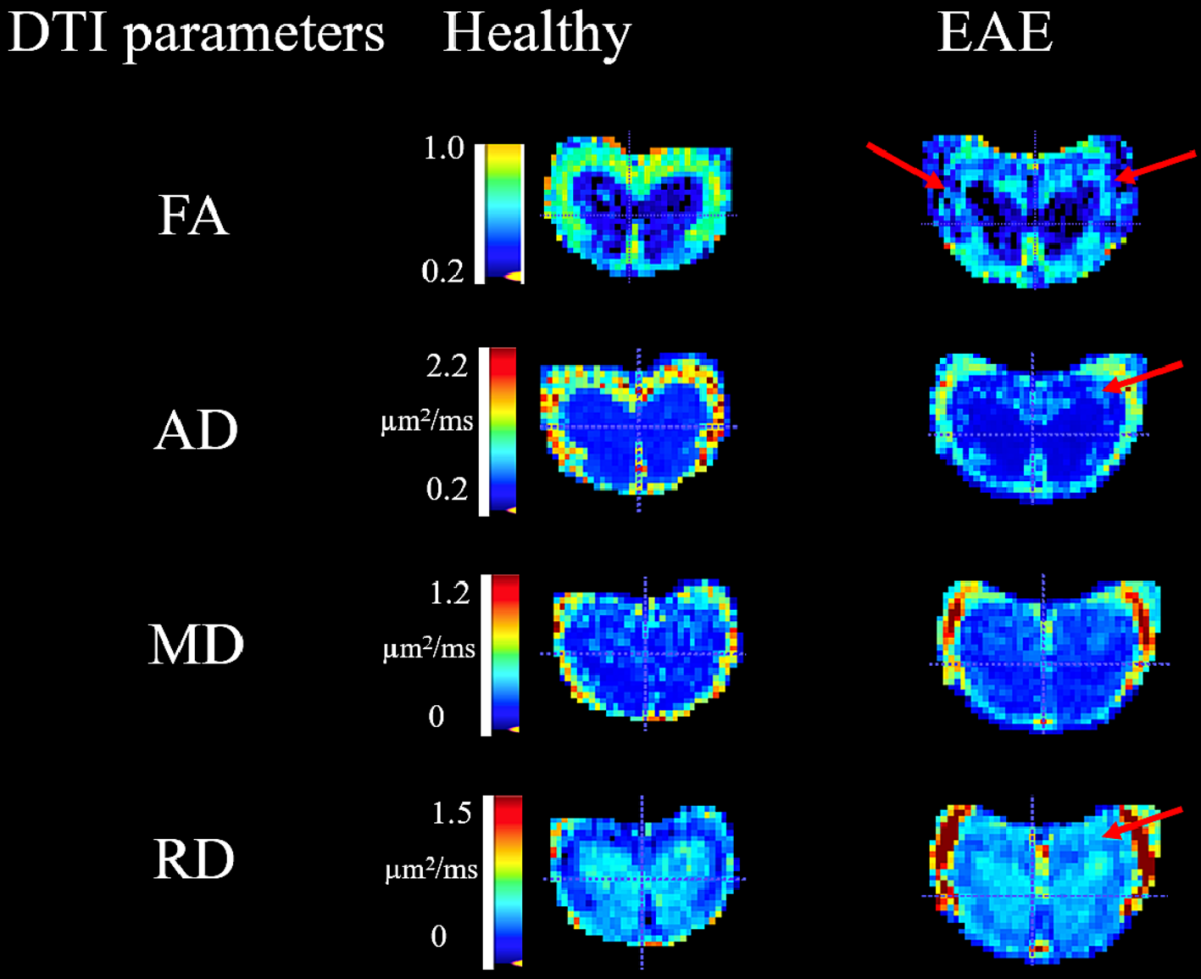
Axial DTI parametric maps at the lumbar level L3/L4. EAE clearly indicated demyelination in the WM, especially by the FA and RD images, as indicated by red arrows. AD, axial diffusivity; DTI, diffusion tensor imaging; EAE, experimental autoimmune encephalomyelitis; FA, fractional anisotropy; MD, mean diffusivity; RD, radial diffusivity; WM, white matter.

DTI parameters FA, AD and RD (but not MD) showed EAE‐induced changes in the WM area. The value of WM FA in healthy mice was approximately 0.6–0.7, as indicated by the green colour (Figure 6). The same region in EAE mice appeared blue (close to GM values), which corresponds to FA of less than 0.4. WM AD was low in the EAE group (~1.00 μm^2^/ms) compared with healthy mice (~1.2 values μm^2^/ms).

**FIGURE 7:**
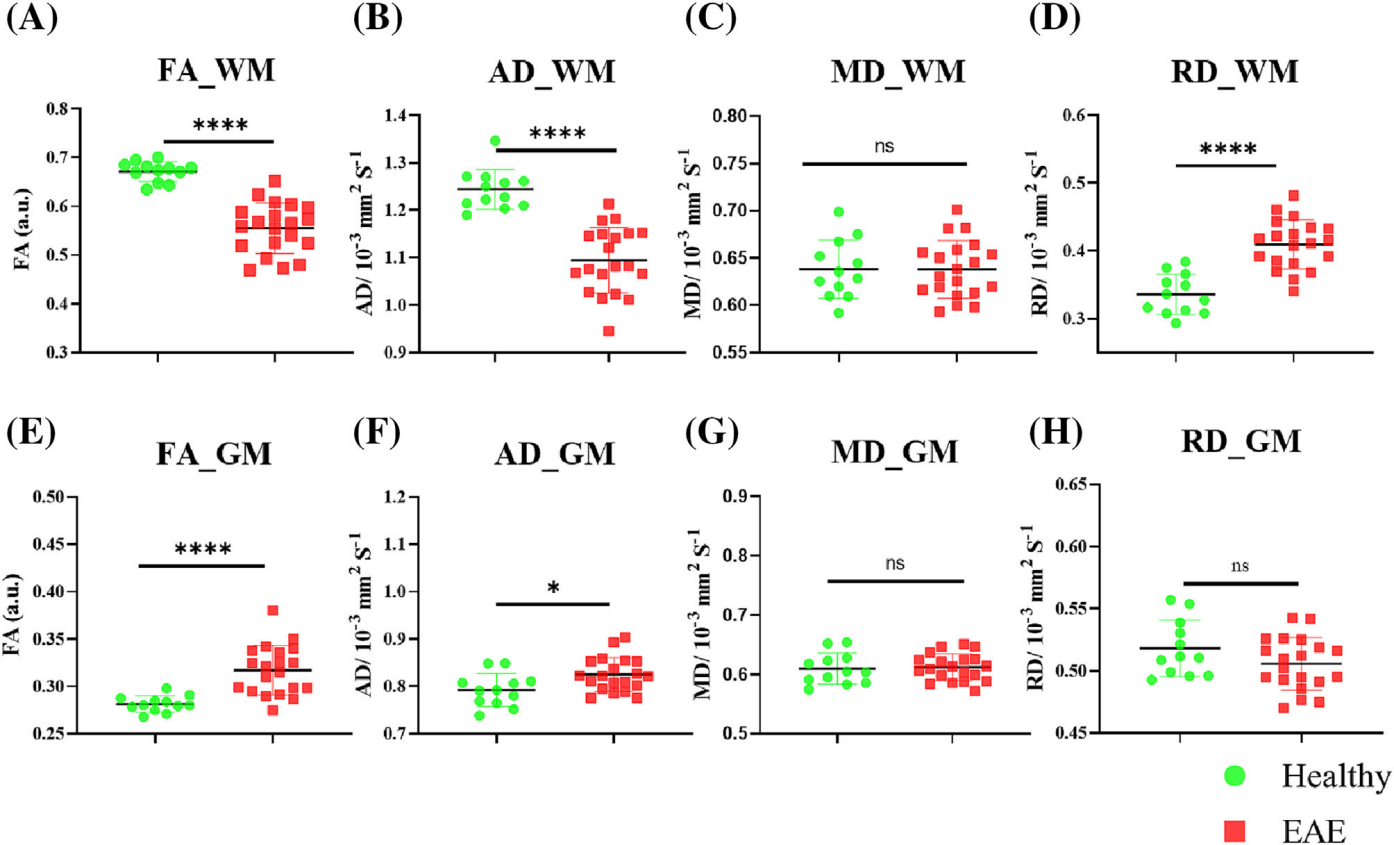
DTI‐derived metrics. Top row: (A) FA, (B) AD, (C) MD and (D) RD changes in the WM. Bottom row: (E) FA, (F) AD, (G) MD and (H) RD changes in the GM. In WM, FA and AD were significantly low, whereas RD was significantly high in EAE. In GM, FA and AD were significantly high in EAE. AD, axial diffusivity; DTI, diffusion tensor imaging; EAE, experimental autoimmune encephalomyelitis; FA, fractional anisotropy; GM, grey matter; RD, radial diffusivity; WM, white matter. *p < 0.05, ****p < 0.0001.

All DTI parameters, other than MD, showed significant changes with various levels of significance (Figure 7). A significant decrease in FA in the WM (0.55 ± 0.05 vs. 0.67 ± 0.02, *p* < 0.0001) and a significant increase in the GM (0.32 ± 0.03 vs. 0.28 ± 0.01, *p* < 0.0001) of EAE mice was observed compared with control WT‐naïve mice. AD in the WM was significantly lower in EAE (1.09 ± 0.07 vs. 1.24 ± 0.04, *p* < 0.0001) and higher in the GM (0.83 ± 0.03 vs. 0.79 ± 0.03, *p* = 0.017). RD was significantly higher in EAE in the WM (0.41 ± 0.037 vs. 0.33 ± 0.03, *p* < 0.0001) and tended to be low in the GM, but not statistically significant (0.51 ± 0.02 vs. 0.52 ± 0.02, *p* = 0.12).

**FIGURE 8:**
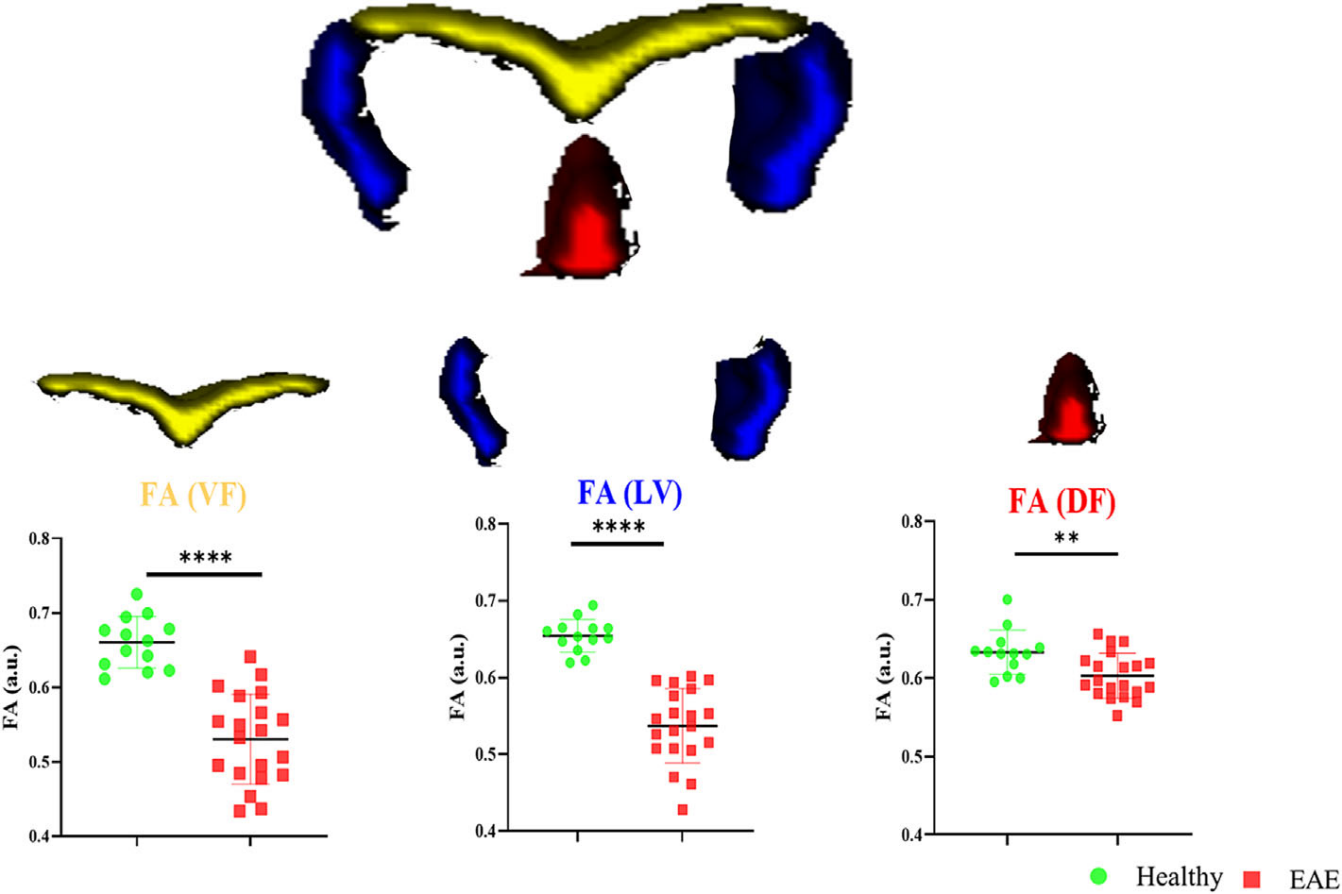
FA values were measured for three WM funiculi. VF, LF and DF are shown in yellow, blue and red, respectively. EAE showed a significant reduction in all three subregions. DF, dorsal funiculus; EAE, experimental autoimmune encephalomyelitis; FA, fractional anisotropy; LF, lateral funiculus; VF, ventral funiculus; WM, white matter. **p < 0.01, ****p < 0.0001.

For the three WM regions, FA was significantly reduced in the EAE group in the VF and LF (*p* < 0.0001). FA within the dorsal region was also reduced, but less significantly (*p* = 0.0058) (Figure 8).

### 3.3 Volumetric changes

**FIGURE 9:**
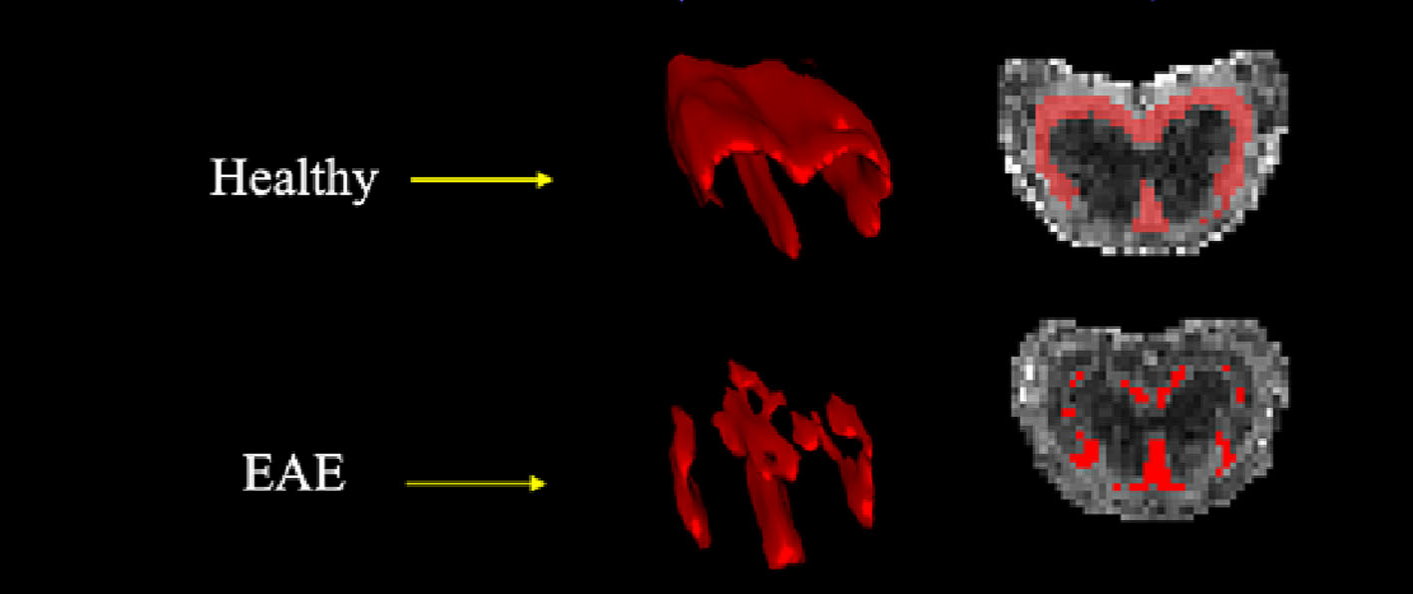
FA was used to threshold GM and WM. FA > 0.5 was used to determine the NAWM, which was significantly reduced in EAE. EAE, experimental autoimmune encephalomyelitis; FA, fractional anisotropy; GM, grey matter; NAWM, normal‐appearing WM; WM, white matter.

**FIGURE 10:**
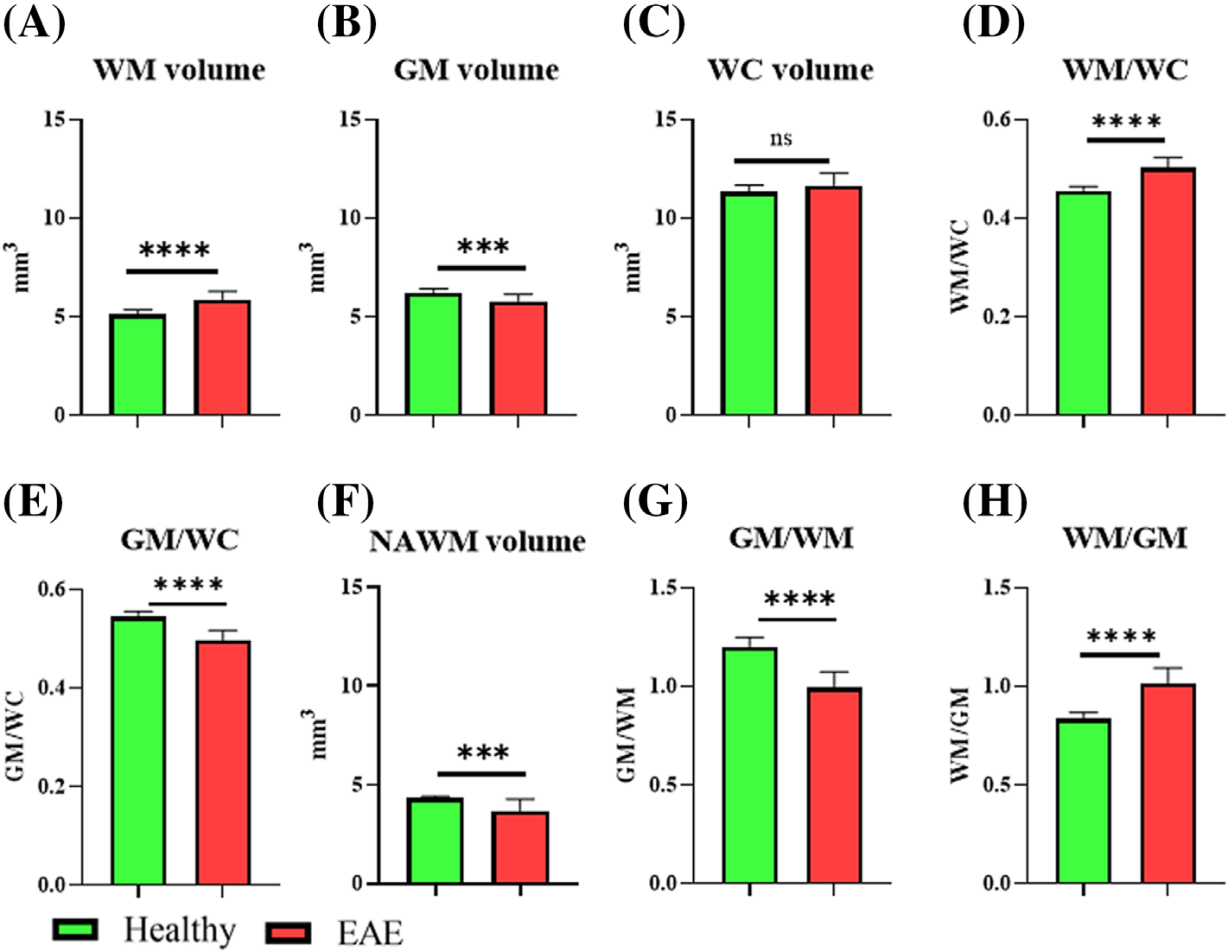
Volumetric changes identified in the lumbar cord. (A) Significant increase of the WM area in EAE. (B) Reduction of the GM area in EAE. (C) WC (GM + WM), where no significant change was observed. (D and E) The differences in WM and GM relative to WC. (F) EAE mice had smaller preserved WM tissue (NAWM) compared with healthy mice. (G and H) The GM/WM and WM/GM ratios. EAE, experimental autoimmune encephalomyelitis; GM, grey matter; NAWM, normal‐appearing WM; WC, whole cord; WM, white matter. ***p < 0.001, ****p < 0.0001.

High‐resolution anatomical imaging of the lumbar whole cord showed a nonsignificant trend of volumetric increase in EAE compared with healthy mice. When the WM and GM volumes were analysed separately, the WM volume was found to be significantly higher in EAE (5.83 ± 0.43 vs. 5.14 ± 0.23 mm^3^, *p* < 0.0001), whereas the GM volume was significantly lower in EAE (5.78 ± 0.35 vs. 6.20 ± 0.22 mm^3^, *p* < 0.001). The volume of NAWM determined using thresholded FA maps showed that NAWM was significantly lower in EAE mice compared with healthy mice (3.6 ± 0.63 vs. 4.3 ± 0.1 mm^3^, *p* = 0.0007) (Figures 9 and 10).

### 3.4 Correlations between EAE scores and MRI measures

Pearson correlation coefficient (r) tests were calculated to measure correlations between MRI parameters and EAE scores and between MRS and DTI metrics. Only moderate and strong correlation findings are reported below. The correlation coefficients are listed in Table 1, and the graph details can be found in Figures S10–13.

**TABLE 1:** Summary table of correlation coefficients between MRI metrics and EAE score.

	MRS					DTI and volumetric measurements									
	NAA/Cr	Cho/Cr	Gly/Cr	Tau/Cr	Lac/Cr	FA_WM	FA (DF)	FA (LF)	FA (VF)	AD_WM	RD_WM	NAWM	WM volume	GM volume	FA_GM
Cho/Cr	−0.4														
Gly/Cr	*−0.5	****0.83													
Tau/Cr	****−0.73	****0.81	****0.85												
Lac/Cr	**−0.65	0.36	*0.51	0.51											
FA_WM	****0.75	**−0.54	**−0.57	****−0.77	−0.23										
FA (DF)	0.41	*−0.5	*−0.45	−0.49	−0.17	***0.67									
FA (LF)	****0.74	**−0.64	***−0.66	−0.82	−0.26	****0.95	****0.73								
FA (VF)	***0.66	*−0.49	*−0.52	−0.67	−0.22	****0.92	**0.55	****0.87							
AD_WM	***0.70	****−0.73	***−0.72	****−0.92	−0.34	****0.84	*0.49	****0.84	****0.77						
RD_WM	***−0.70	0.39	*0.42	**0.59	0.15	****−0.94	***−0.7	****−0.9	****−0.87	**−0.64					
NAWM	**0.57	−0.33	−0.41	−0.59	0.02	****0.87	*0.5	****0.82	****0.87	***0.71	****−0.82				
WM volume	**−0.53	**0.59	**0.54	0.7	0.35	****−0.77	**−0.57	***−0.72	**−0.62	****−0.78	**0.64	*−0.43			
GM volume	0.41	**−0.55	*−0.51	−0.55	0.19	**0.57	0.38	***0.65	**0.55	*0.49	**−0.54	**0.61	−0.21		
FA_GM	−0.23	**0.57	0.47	*0.48	0.2	−0.28	−0.14	−0.35	−0.38	−0.4	0.23	−0.22	0.14	−0.34	
EAE score	****−0.75	****0.80	****0.75	****0.90	*0.50	****−0.87	**−0.51	****−0.88	****−0.84	****−0.84	****0.76	****−0.66	****0.76	**−0.53	***0.60
				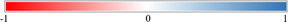											

Abbreviations: AD, axial diffusivity; Cho, choline; Cr, creatine; DF, dorsal funiculus; DTI, diffusion tensor imaging; EAE, experimental autoimmune encephalomyelitis; FA, fractional anisotropy; Gly, glycine; GM, grey matter; Lac, lactate; LF, lateral funiculus; MRI, magnetic resonance imaging; MRS, magnetic resonance spectroscopy; NAA, *N*‐acetyl aspartate; NAWM, normal‐appearing WM; RD, radial diffusivity; Tau, taurine; VF, ventral funiculus; WM, white matter.

*p ≤ 0.05; **p ≤ 0.01; ***p ≤ 0.001; ****p < 0.0001; no *p > 0.05.

#### 3.4.1 EAE scores versus MRS metabolites

There was a strong negative correlation of EAE score with NAA (r = −0.75, *p* < 0.0001) and positive correlations of EAE score with each of Cho/Cr (r = 0.80, *p* < 0.0001), Gly/Cr (r = 0.75, *p* < 0.0001), Tau/Cr (r = 0.90, *p* < 0.0001) and Lac/Cr (r = 0.50, *p* = 0.048).

#### 3.4.2 EAE scores versus DTI parameters

EAE scores were found to be strongly correlated with WM DTI metrics. EAE scores were negatively correlated with FA (r = −0.87, *p* < 0.0001) and AD (r = −0.83, *p* < 0.0001) and positively with RD (r = 0.75, *p* < 0.0001). In the three subregions of WM, the LF showed the strongest correlation with the EAE score (r = −0.88, *p* < 0.0001). In GM, only FA showed a strong positive correlation with the EAE score (r = 0.60, *p* < 0.0001).

WM volume change showed a strong positive correlation with EAE score (r = 0.76, *p* < 0.0001), whereas GM volume change showed a moderate negative correlation (r = −0.53, *p* = 0.002).

#### 3.4.3 Correlations between MRS and DTI metrics

MRS voxels were primarily located in the lumbar GM region, whereas DTI metrics used ROIs that covered both GM and WM structures. It is interesting that although MRS and DTI measurements were not taken from adjoining GM/WM regions, there were strong and medium correlations between NAA/Cr and Cho/Cr MRS measures with DTI FA, AD and RD.

### 3.5 Immunohistochemistry

**FIGURE 11:**
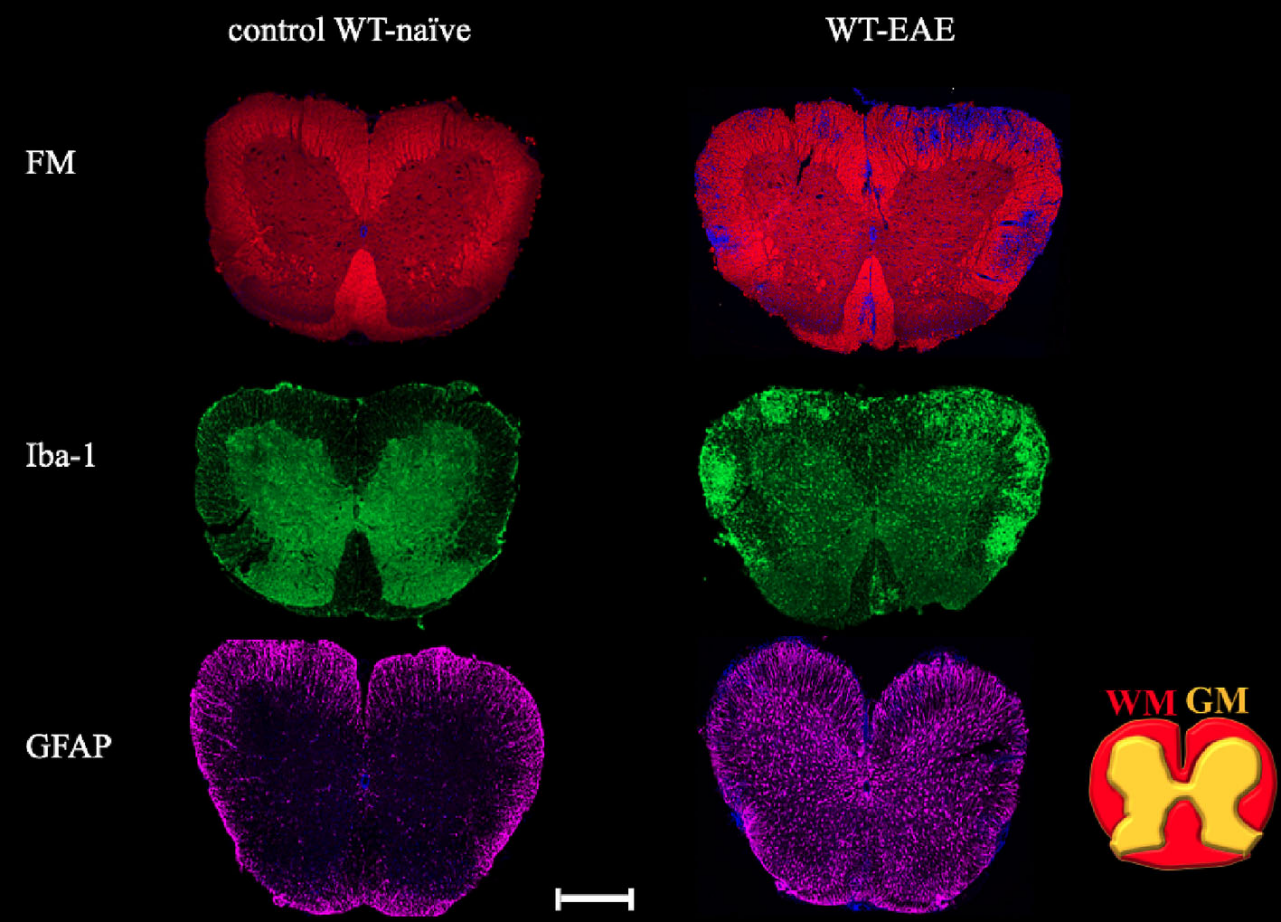
Immunofluorescence staining of EAE pathology in the mouse lumbar spinal cord. FM staining (red) showed axonal myelination in WM and GM. EAE showed demyelinated areas predominantly in the WM, where DAPI nuclear staining background (blue) appeared. Activated microglia cells were demonstrated in Iba‐1 (green signal) in EAE in GM and WM, particularly in areas that showed demyelination. GFAP showed aggregation of astrocytes in GM (purple signal). Images were taken from adjacent slices. Scale bar indicates 600 μm. DAPI, 4′,6‐diamidino‐2‐phenylindole dihydrochloride; EAE, experimental autoimmune encephalomyelitis; FM, FluoroMyelin; GFAP, glial fibrillary acidic protein; GM, grey matter; Iba‐1, ionised calcium binding adaptor molecule 1; WM, white matter.

Significant demyelination in the EAE mouse lumbar spinal cord was observed by IHC. Demyelination areas were indicated by the disappearance of FM red staining and the appearance of DAPI blue signal, predominantly within the WM. These demyelination regions were colocalised with Iba‐1 staining (green), which was observed in both WM and GM. GFAP staining showed astrogliosis (purple) in EAE, mainly in the GM regions (Figure 11).

## 4 DISCUSSION

Here, we have evaluated the ability of multiparametric MR measures, including MRS, DTI and high‐resolution structural MRI, to reveal EAE‐induced changes in the lumbar spinal cord. Enhanced detection of the pathology by MRI was enabled with the use of cryo‐coil technology and higher disease severity in the progressive EAE model. A strong inflammatory reaction was confirmed by immunofluorescence microscopy showing extensive demyelination, microgliosis and astrogliosis. Changes in MRS Cho and NAA metabolites and DTI RD and AD metrics appeared to be highly sensitive MRI biomarkers for demyelination and axonal damage.

### 4.1 Changes in spinal cord metabolites

The assessment of metabolite changes in the lumbar GM region is important because we observed a strong increase in astrocyte and microglia/macrophage activation in the EAE mice (Figure 11). Our observations agreed with MRS studies of human spinal cord GM in MS.^9^, ^10^, ^15^, ^16^, ^17^, ^18^, ^19^ To the best of our knowledge, this is the first study reporting MRS‐based evaluation of metabolites in the CNS, particularly the lumbar spinal cord, in an EAE mouse model.

In healthy animal groups, values of metabolite concentrations measured relative to Cr were similar to those previously reported^27^ with the exception of Gly and m‐Ins. Based on our phantom experiments (Figures S14 and S15) and another study,^44^ it is possible that the previous study^27^ had misassigned the m‐Ins peaks as Gly at 3.65 ppm.

Comparison between control and EAE groups showed no significant difference in the Cr, indicating a similar level of energy metabolism.^18^ This finding is consistent with that found in human MS in the brain^11^, ^12^ and in the spinal cord.^18^, ^19^

NAA was significantly reduced in the EAE group compared with healthy mice (Figure 4). This finding is similar to that reported in human spinal cord MS,^9^, ^15^, ^18^, ^19^ indicating the presence of neuronal loss and axonal damage. The reduction in NAA was also associated with a high EAE score.

Cho was significantly increased in EAE mice (Figure 4). A previous spinal cord MS study^10^ suggested that an increase in Cho reflected an increase in membrane phospholipid turnover as a result of myelin breakdown. Demyelination was clearly seen in our EAE mice using FM staining. Our findings are largely consistent in terms of NAA and Cho changes with human MS in the brain^11^, ^13^ and spinal cord studies.^16^, ^17^ Other MS spinal cord studies found no significant change in Cho.^9^, ^15^, ^18^, ^19^ Cho was also found to correlate with EAE score, and it was stronger in animals with an EAE score of 3 (Table 1).

An increase of m‐Ins in the lumbar region of EAE mice was originally expected as it was found to be significantly high in MS in both the spinal cord^16^, ^17^, ^18^, ^19^ and the brain,^13^, ^45^, ^46^ but this was not detected in our study. m‐Ins peaks were initially detected in all control animals, but in most EAE animal data, m‐Ins signal detection was not reliable as the SD of peak fit was high. Other studies have reported no change in m‐Ins.^15^

Instead of an m‐Ins increase, a significant increase in Gly was observed in EAE mice (Figure 4). This observation is unique as it has not been reported in MRS studies of human MS and EAE. A recent study using liquid chromatography high‐resolution mass spectrometry detected a significant increase in Gly in the CSF of patients with secondary progressive MS.^47^ In addition, an animal study by Musgrave et al.^48^ using high‐performance liquid chromatography found that mice with EAE exhibited higher Gly in spinal cord compared with healthy mice. The increase of Gly is presumably due to the body's response to alleviate oxidative stress.^49^, ^50^ Oxidative stress has been associated with microglia/macrophage activity and increased severity of demyelination.^51^ In our EAE mice, microglia/macrophage infiltration was confirmed by Iba‐1 staining (Figure 11). Tau was found to be significantly higher in EAE compared with healthy mice (Figure 4). This is in agreement with studies of human MS, as Tau was found to be higher in plasma allopregnanolone of patients with relapsing‐remitting MS.^52^ Similar to Gly, Tau has not been reported in any MRS studies in MS.

For disease characterisation, overlapping metabolite peaks can make it difficult to determine whether spectral changes were due to an increase or decrease in specific metabolites and whether their changes were synchronous or contradictory.^53^ Our spectroscopy simulation and phantom experiments showed that Gly and m‐Ins peaks overlapped at similar resonance frequencies (Figure S14). Gly appeared as a singlet at 3.56 ppm and m‐Ins as a multiplet at 3.45–3.65 ppm (Figure S15).^44^, ^53^ Therefore, both Gly and m‐Ins may have increased in our EAE mice, but MRS was only sensitive to detect the increase in Gly. The peak increase at 3.56 ppm may be dominated by the strong signal from the Gly singlet, and not sensitive to m‐Ins. In brain spectra, we found the exact opposite results in terms of Gly/m‐Ins detection, because Gly showed high SD (low concentration) and m‐Ins was detected (high concentration) in all brain spectra.

Lac was significantly increased in EAE mice. Lac is a byproduct of pyruvate reduction in anaerobic glycolysis, which is believed to function as an alternative cellular energy source during neuroinflammation including MS, where high levels of neural activity have occurred.^54^, ^55^ In addition, Lac is known to be synthesised by astrocytes,^56^ and the increase in serum Lac was reported to be positively correlated with disability in patients with MS.^57^ Our data confirmed GFAP staining for astrogliosis in the EAE (Figure 11), and the increase in Lac was positively and moderately correlated with EAE score.

### 4.2 Changes in spinal cord tissue microstructure

Progressive EAE pathology in the mouse lumbar spinal cord is characterised by FA and AD decreases and RD increases in the WM, but not in the GM regions (Figure 7). The decrease in FA was larger in the VF and LF compared with the dorsal region, as confirmed by IHC. The observed significant FA WM change in the LF also agreed with a previous report.^58^ FA was strongly negatively correlated with EAE score, similar to observations in MS^2^ and the EAE mouse model.^24^

It has been suggested that reduced FA indicates demyelinated fibre tracts or axonal loss, as any increase in RD or decrease in AD will lead to a decrease in FA.^28^ FA was reported to be significantly low in both MS lesions^59^ and NAWM.^2^

The presence of EAE‐induced axonal damage was confirmed by a strong correlation between the reduction of AD and the reduction of NAA and a very strong negative correlation between the decrease of AD and the increase in EAE score (Table 1). Our observations supported the findings of other mouse studies with different degrees of axonal damage in the EAE mouse model.^28^, ^60^ There has been agreement that a low AD represents a good biomarker for axonal degeneration or deficits. AD quantifies the movement of water molecules along the axon fibres, and therefore, its reduction reflects the loss of coherent organisation of the axon and physiological mechanisms associated with axon damage and degeneration.^28^, ^61^

RD was significantly increased in EAE mice (Figure 7). RD quantifies the radial movement of water molecules between the axonal tracts, whereby areas with healthy myelinated axon bundles typically have low RD due to restricted radial water diffusion. High RD has been suggested as a biomarker for the loss of myelin membrane integrity, which allows for an increase in radial water diffusion. Indeed, clear consequential relationships between RD, demyelination and axonal loss have been reported,^24^, ^28^ resulting from complex microstructural changes (e.g., gliosis and cellular infiltration) in neurodegenerative disease. RD changes observed in this study were similar to those observed in human MS.^2^ Furthermore, RD increases were strongly and positively correlated with EAE score (Table 1); such patterns were also found in another EAE study, but in only a few ROIs along the periphery of ventrolateral WM.^24^ The increase in WM RD was negatively correlated with a decrease in NAA. As the MRS voxel predominantly contained the GM structure, such a correlation may highlight related injury of neuronal bodies in the GM and their axonal projections in the WM.

MD did not display significant changes in WM and GM, whereas FA increased in the GM. This pattern was different to that reported in human MS^2^; however, this observation may be explained by the underlying complex disease activity. IHC data showed a marked increase in cellular density, composed of activated microglia/macrophages and astrocytes in both WM and GM tissue together with demyelination. The increase of FA in GM could be a result of increased restricted diffusion from glial cell body and processes or axonal degeneration due to fibre transection in remote focal lesions.^62^

### 4.3 Volumetric changes in the spinal cord

EAE mice exhibited GM atrophy and WM hypertrophy in the lumbar spinal cord (Figure 10). GM atrophy has been observed in the brain of EAE mice^23^, ^29^ and was characteristically observed as neuronal loss in MS.^63^ GM volume was moderately and negatively correlated with EAE score (Table 1), which is in line with a previous EAE study^29^ and an MS study where the loss of brain GM tissue was shown to be correlated with the degree of disability.^64^

By contrast, WM hypertrophy could result from inflammatory processes, including tissue swelling and aggregation of immune cells such as microglia/macrophages, as confirmed by IHC. The increase in WM volume was strongly and positively correlated with EAE score, which also supports previous findings in the brain of EAE mice.^22^ Whole brain hypertrophy was detected at the peak of EAE score (18 days postimmunisation); however, at the more chronic phase (66 days postimmunisation), the whole brain (WM and GM) volumes were reduced. By comparison, individuals with relapsing‐remitting MS during relapse (with active lesions) have been shown to exhibit brain hypertrophy, presumably as a result of blood–brain barrier damage, infiltration of inflammatory cells from the periphery and gliosis.^65^ However, in the absence of active lesions, MS brain was steadily progressing with atrophy, a process that was independent of age, sex and treatment.^65^

### 4.4 Preclinical methods translation for MS

We demonstrated the benefits of using a multiparametric study to measure MS‐like pathology in a preclinical animal model providing a more complete picture of the disease pathology. The use of cryoprobe detection at 9.4 T allowed observation of new MRI biomarkers such as increased Tau and Gly and increased FA in the GM. Translation of these methods to clinical systems for human investigation can be challenging because of a potentially lengthy acquisition time and lower SNR in typical (1.5–3 T) human scanners. However, they should be pursued because they will assist clinical diagnosis and efficacy of treatments. Cryoprobe technology is currently not available in human scanners, but increased MR sensitivity may be obtained using a dedicated spinal cord coil and imaging at ultrahigh field using 7‐ and 9.4‐T scanners.^66^, ^67^

### 4.5 Limitations and future recommendations

In a longitudinal study for 18 months, patients with relapsing‐remitting‐MS have shown an increase in MD and a decrease in FA in normal appearing GM.^32^ Hence, it is plausible to investigate temporal changes in EAE disease to identify MRI biomarkers for various disease phenotypes. Future studies are warranted to determine whether the MRI‐based pathobiological changes in EAE mice were related to MOG_35–55_ or adjuvants by using sham mice immunised with CFA and/or pertussis without MOG_35–55_. Neurofilament light, a recognised biomarker for axonal damage,^68^ can also be used to validate the MRI‐based changes in EAE mice. We also recommend acquiring an additional spectrum with a long TE to ascertain the increase of Lac from the lipid signal.

## 5 CONCLUSION

MRS and DTI measures were found to be sensitive to EAE pathology and highly correlated with EAE score. A typical feature of EAE is demyelination, which was indicated by an increase in Cho and RD in EAE mice compared with healthy mice. Demyelination was confirmed by IHC in FM staining. Neuronal loss and axonal damage were also indicated by a reduction in AD and NAA. Aggregation of cells such as astrocytes (forming scar tissue postinflammation) in GM areas was indicated by an increase in FA. EAE was also accompanied by atrophy in GM and hypertrophy in WM.

The observations described here provide the basis for a set of MR biomarkers for monitoring the status and progression of EAE pathology in vivo in this important animal model of MS. Application of this approach to demonstrate the efficacy of novel MS drug candidates and their mode of action is the aim of our ongoing work. While translation of these measures to application in human spinal cord in MS has not yet been demonstrated, the advent of ultrahigh‐field human MR technology (7 T and above) promises to bring the required sensitivity to within reach of clinical diagnostic and drug trial regimes.

## Supporting information


**Data S1.** Supporting Information
