# Supplementary material for: Multiparametric magnetic resonance imaging for detection of pathological changes in the central nervous system of a mouse model of multiple sclerosis in vivo
Source: NMR Biomed. 2023 May 18;36(10):e4964. doi: 10.1002/nbm.4964 (PMC10909458; doi:10.1002/nbm.4964)
Supplement: Supplementary file 1 — Data S1. Supporting Information [file NBM-36-e4964-s001.docx]

SUPPLEMENTARY MATERIAL 1: EAE scoring sheet.

Table S1. 5-point scoring system.

| Score | EAE Signs | Description |
| --- | --- | --- |
| 0.0 | Normal Behaviour | Normal gait: Tail has tone when touched with finger |
| 0.5 | Distal Limp Tail | Only tip of the tail is limp, this is usually identified by a hook shape formed at its distal part. The animal is still able to keep the tail upright during walking or when picked up by finger. |
| 1.0 | Complete Limp Tail | The whole tail droops and does not wrap around cylindrical object or finger. The animal is unable to keep the tail upright once picked up by finger. The animal still appears to have normal gait. |
| 1.5 | Limp Tail +  Slight Hindlimb Weakness | Subtle hindlimbs weakness is identified by their slight outwards protrusion during gait. Animal walking is only slightly wobbly. |
| 2.0 | Hindlimb Weakness +  Partial paralysis of hindlimbs | The animal clearly shows waddling gait (usually identified by whipping of tail) due to hindlimbs weakness and/or partial paralysis of hindlimbs particularly their toes. At this stage or hereafter, the animal has difficulty to return on its feet when laid on its back. |
| 2.5 | Distinct unilateral hindlimb paralysis | Usually one hindlimb is paralysed, which is identified by continuous or intermittent dragging during walk. At this stage, some animals may show severe jerky walk without dragging their hindlimbs. |
| 3.0 | Complete bilateral hindlimbs paralysis | Both hindlimbs are dragged and the animal usually moves with the strength of their forelimbs. At this stage, some animals might develop one hindlimb and one forelimb paralysis. The animal is still able to slightly move around the cage. At this stage, food gel packs are placed on the cage floor. |
| 3.5* | Bilateral hindlimb paralysis +  Forelimb paralysis | Both hindlimbs and one forelimb are paralysed. The animal is unable to move around the cage. |
| 4.0 | Quadriplegia | Complete paralysis of hind limbs and forelimbs. Hindlimbs are dragged behind and forelimbs do not respond to toe pinch. |
| 5.0 | Death |  |

3.5* = In this project we euthanize mice when they reach a score ≥ 3.5 as per animal ethical committee conditions.

**SUPPLEMENTARY MATERIAL 2: Mouse positioning for lumbar spinal cord imaging using a cryoprobe**


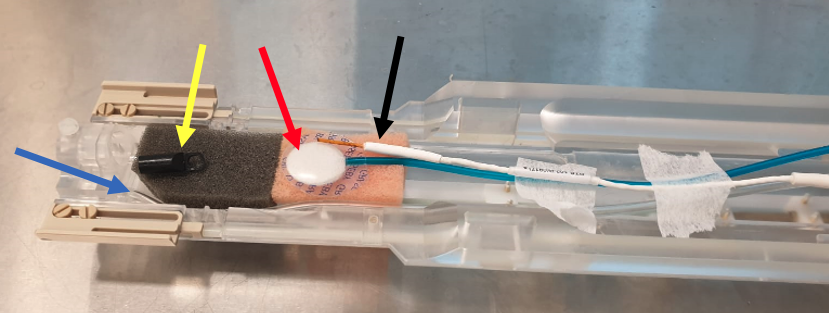

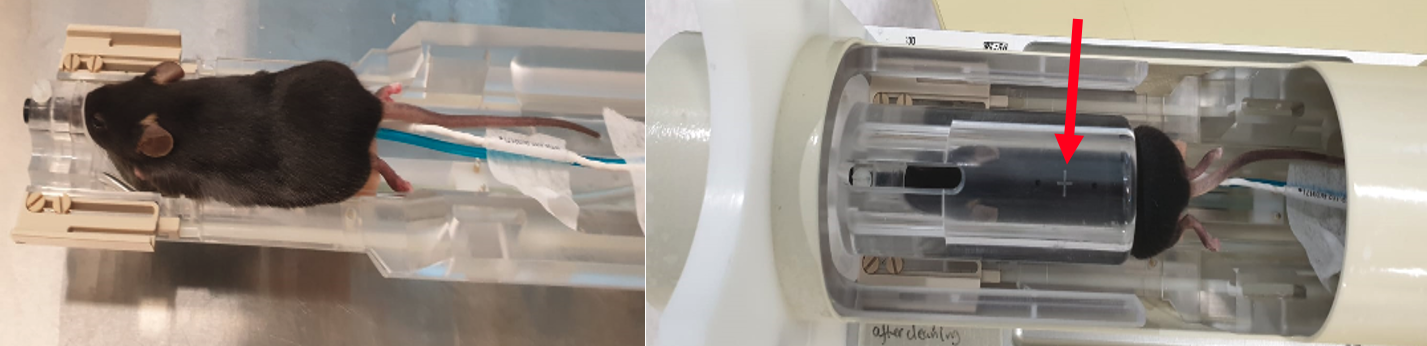

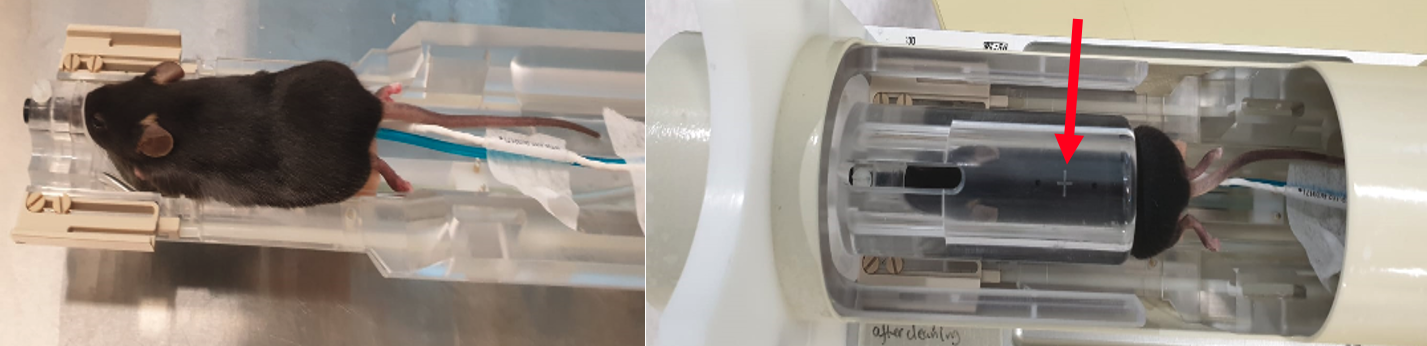


A

B

C

**Figure S1. Preparation for spinal cord imaging using Bruker mouse head quadrature Tx/Rx cryoprobe surface coil.** (A) Animal bed preparation. Blue arrow points to the anaesthesia supply tube. Yellow arrow points on the tooth hook for mouse positioning. Red and black arrows indicate the respiration and temperature sensors respectively. (B) Mouse setup on the cryoprobe bed. (C) Animal position was checked using the mock cryoprobe, the lumbar region placed in the centre of the coil as indicated by red arrow on the + mark.

**SUPPLEMENTARY MATERIAL 3: Comparison of lumbar MR spectra acquired at two TEs**

Figure S2. Comparison of lumbar MR spectra acquired at two TEs. Measured Gly/Cr was lower at TE = 30 ms compared to TE = 14 ms.

**SUPPLEMENTARY MATERIAL 4: Metabolite spectral simulations and quantification**

**Simulation method**

Quantitation of some metabolites, such as Gly, m-Ins, Glu, NAA and Lac is problematic due to signal overlap. To understand the components and deconvolution of all metabolite peaks in a spectrum, simulated spectra were generated using *Mathematica* software (version 12.1). The Bloch equations were used to simulate the spectrum at 9.4T (400.13 MHz) with the assumptions that water was fully suppressed and T_2_ relaxation of metabolites was very long (2000 ms). Chemical shift values were obtained from the Biological Magnetic Resonance Data Bank (<http://www.bmrb.wisc.edu/>), and metabolite molarity was as described within the "LCModel & LCMgui user's manual". Each of the metabolites were simulated individually and then summed to generate the simulated spectrum (Figure S3 A and B).

To ensure the confidence in metabolite quantitation, each spectrum was assessed using (*i*) standard deviation of the peak fit, (*ii*) FWHM, (*iii*) flat baseline and (*iv*) SNR. The metabolites Cr, NAA, Cho, Gly, Lac and Glx typically achieved the standard deviation peak fitting of ≤15%, except m-Ins. Any spectra with unsuppressed water FWHM > 28 Hz, without a flat baseline and SNR ≤ 9 were also excluded. There were 25 out of 36 samples (69.4%) that fulfilled the quality control criteria; these were comprised of EAE mice n= 13 and healthy mice n=12. Figure S4 C shows an example of typical spectra that were included for analysis.

| A 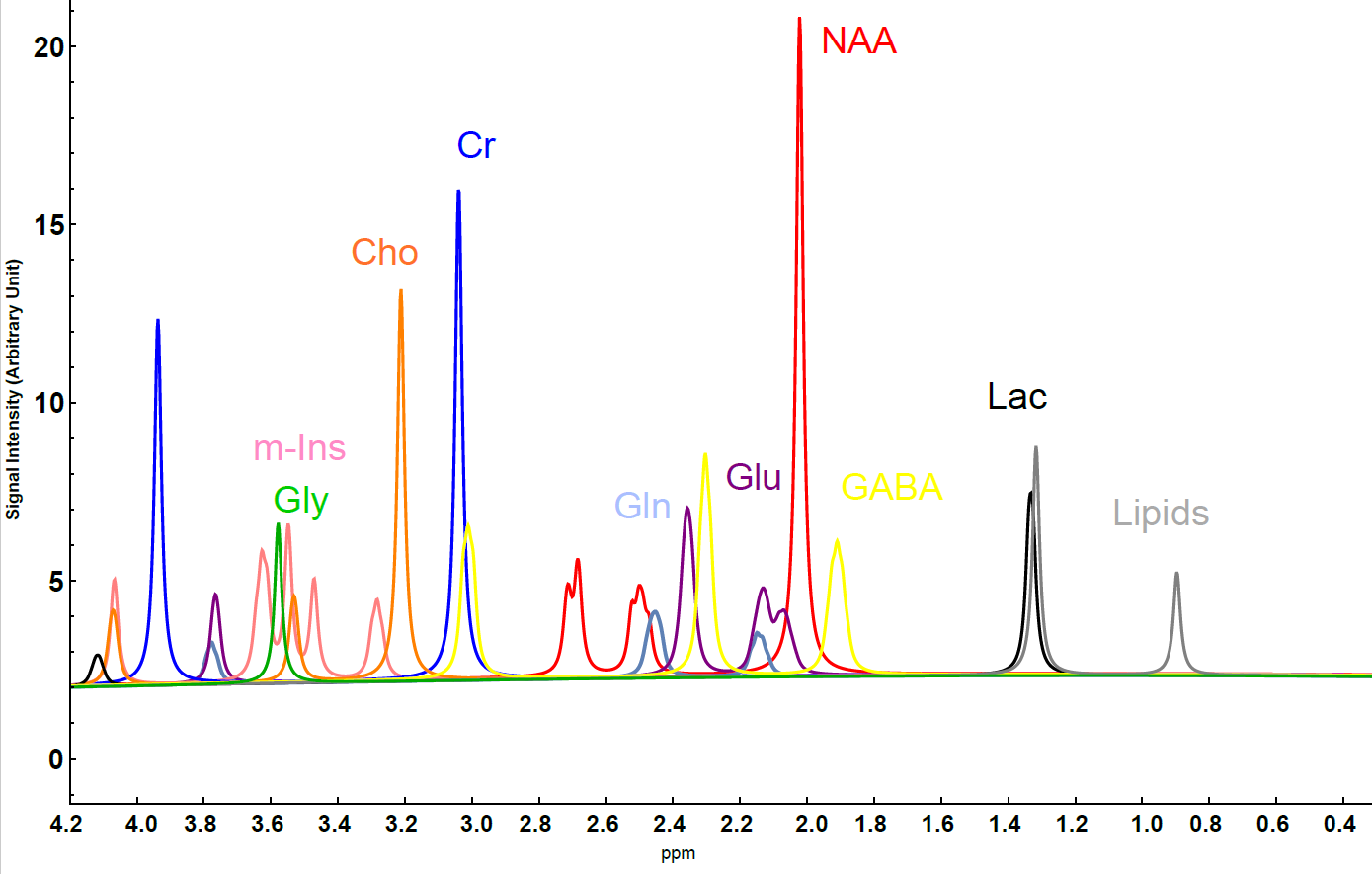 |
| --- |
| B 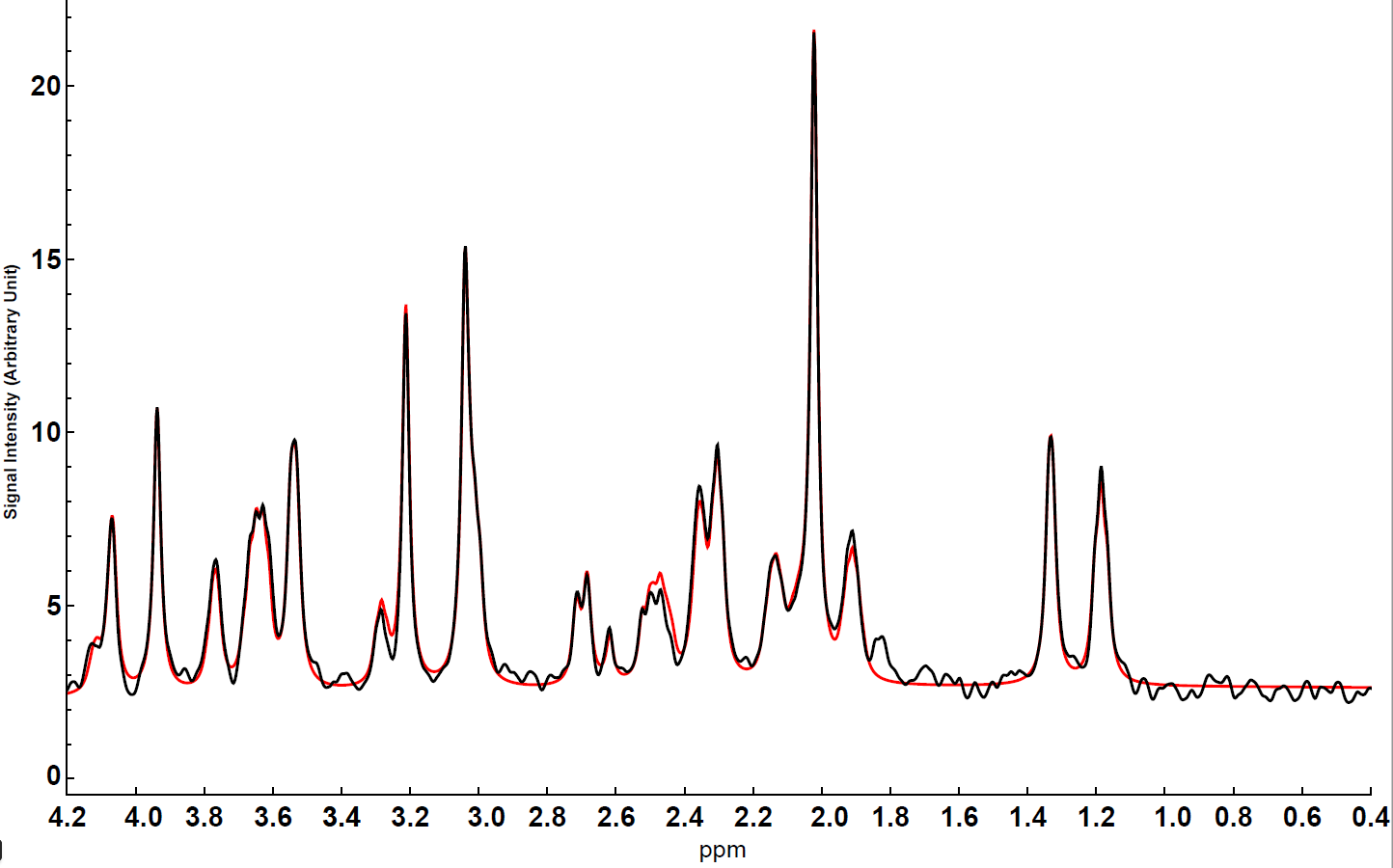 |
| C 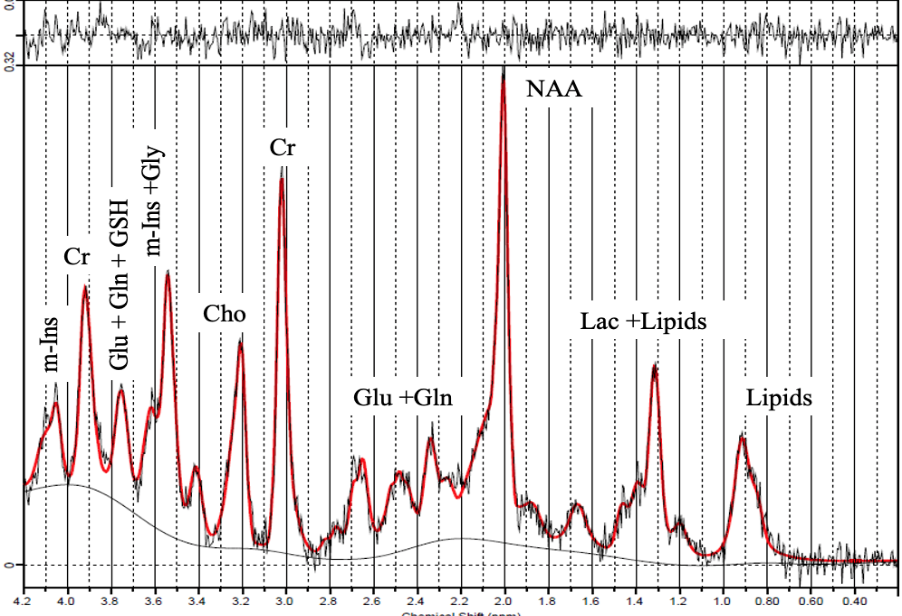 |

Figure S3. MR spectroscopy simulation of CNS metabolites with (A) and without colour coded (B), and typical acquired spectrum (C) of the lumbar spinal cord acquired at 9.4T using a cryoprobe. Several metabolite peaks have overlaps such as Gly with m-Ins, NAA with Glu, and Lac with Lipids. LCModel fitting of the metabolite peaks is shown in red, with the fitting residual shown at the top.

LCModel measurement was initially performed using the program default setting in which fitting was performed over 0.2 – 4.0 ppm and Gly was not included in the basis set. This approach, however, failed to consistently detect m-Ins, particularly in the EAE group. For the few samples whose m-Ins were detected by LCModel (healthy n = 10 and EAE n = 3), each group had a high standard deviation and hence no statistical difference was detected. To test whether LCModel would require Gly to be included to achieve good fit, Gly was added to the basis set and the spectra were reanalysed with an increased spectral range (0.2 to 4.2 ppm). This approach achieved good spectral fit for all analysed data, but m-Ins remained unresolved (Figure S4 D).

| A  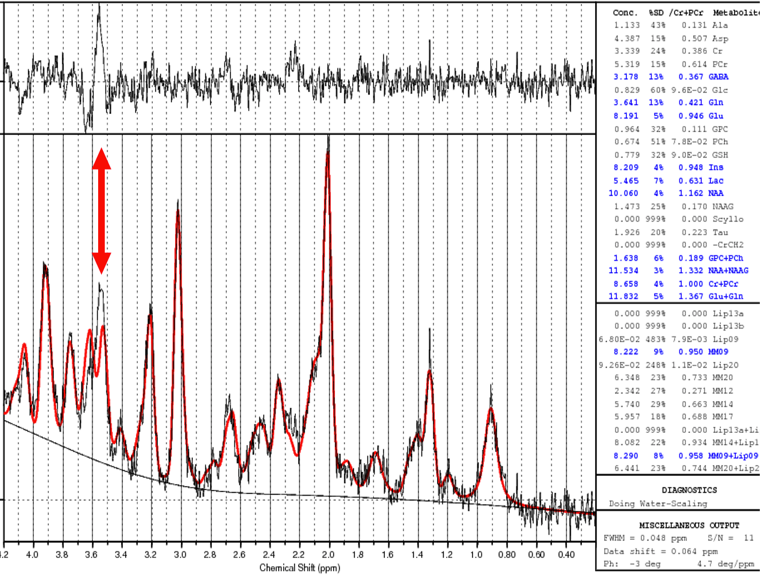 | C  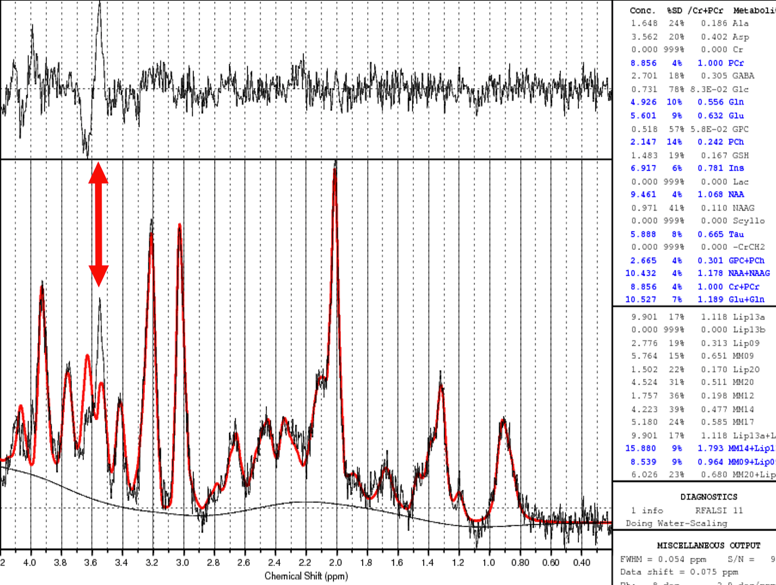 |
| --- | --- |
| B  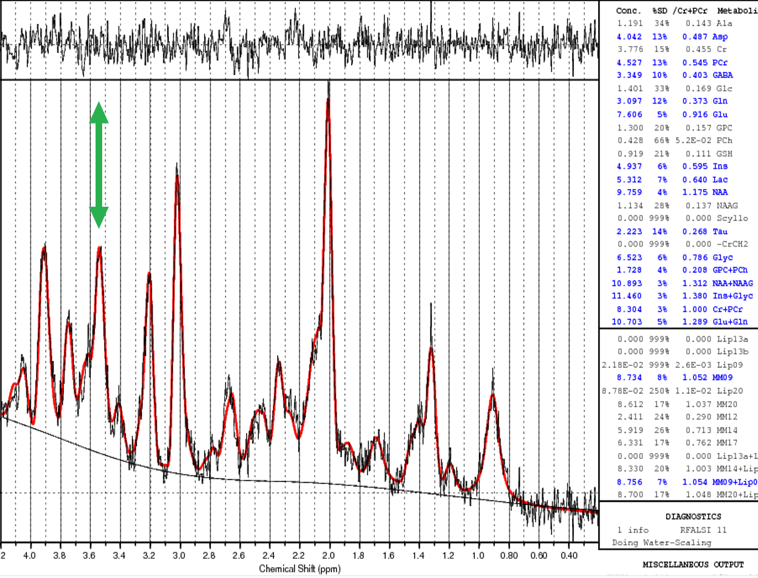 | D  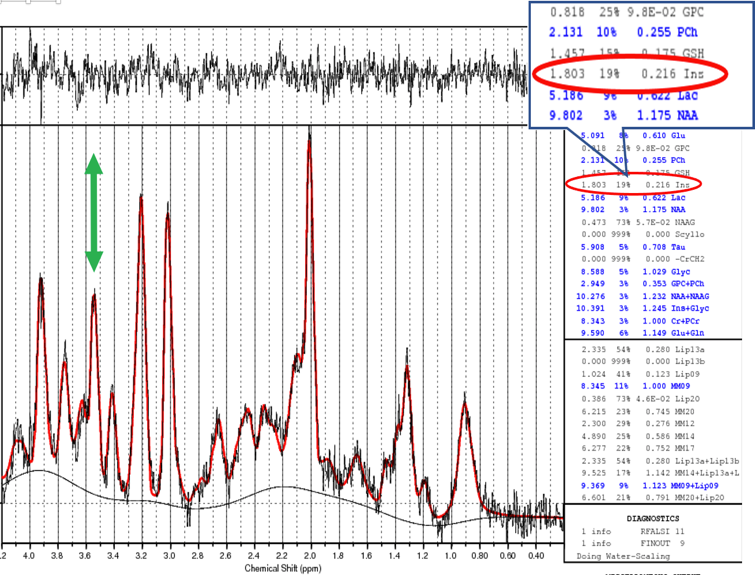 |

Figure S4. LCModel fitting of lumbar spinal cord MRS data. Fitting was performed with data that had fulfilled the selection criteria. The fitted spectra (red line) were overlaid onto the experimental data (black line). (A) spectrum showing poor fitting and more residuals (red arrow) for healthy mouse processed by default settings (Gly was not included). (B) Fitting of experimental data in (A) with Gly included, showing good fitting with less residual fit (green arrow). (C) LCModel fitting of a spectrum taken at the lumbar spinal cord of EAE mouse, the default setting result in poor fitting and high residuals (red arrow). (D) Fitting of experimental data in (C) with Gly included, showing good fitting with less residual fit (green arrow). Although the fitting (after adding Gly to data sets) and SNR were good, m-Ins still not detected in EAE mice (red circle).

**SUPPLEMENTARY MATERIAL 5: LCModel fitting of the spectra of the mouse brain**


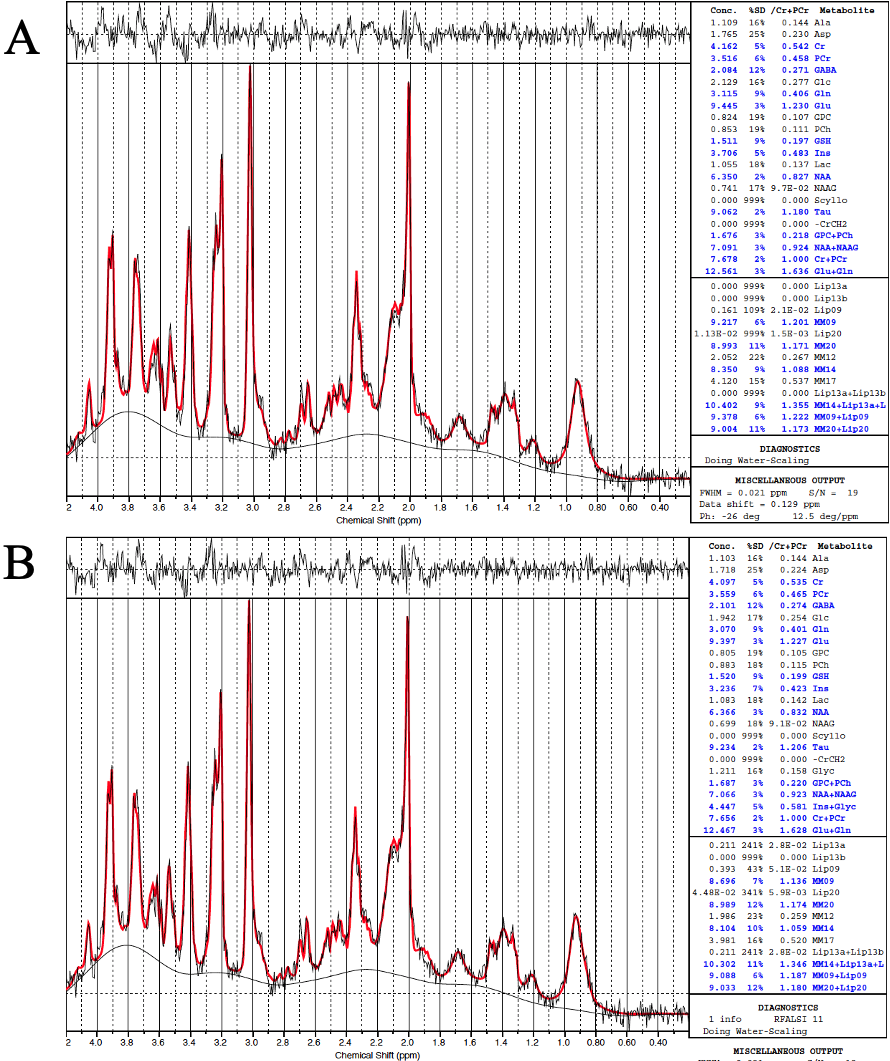


Figure S5. MRS of the mouse brain fitted with or without Gly in the basis set. MRS were acquired at TE 14 ms. There was no difference in LCModel fitting quality for the calculations performed (A) without or (B) with including Gly in the basis set. Gly remained outside the criteria used for detection (SD > 15%) and at very low concentration (Gly/Cr = 0.158) (red arrow).

**SUPPLEMENTARY MATERIAL 6: Retraining of SCT DL segmentation**


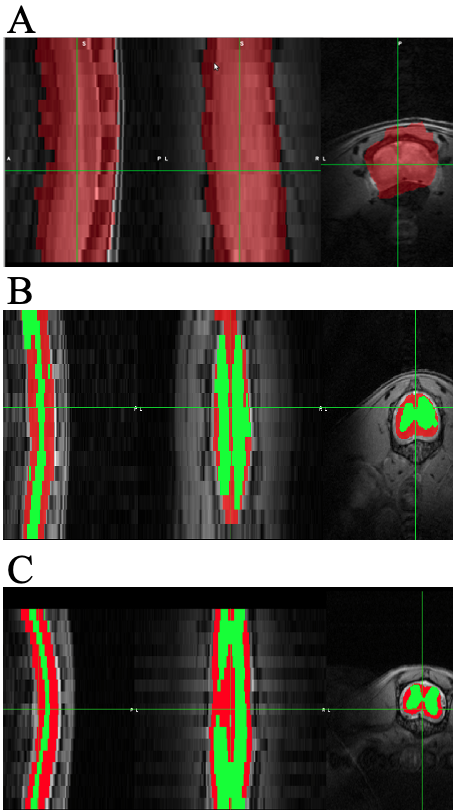

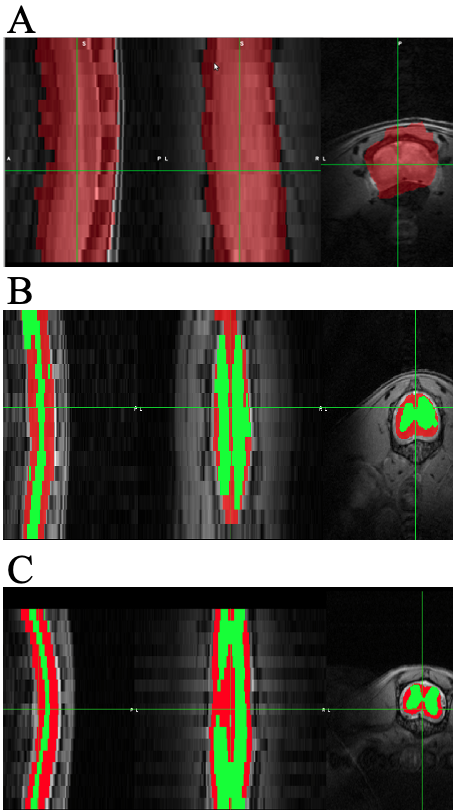

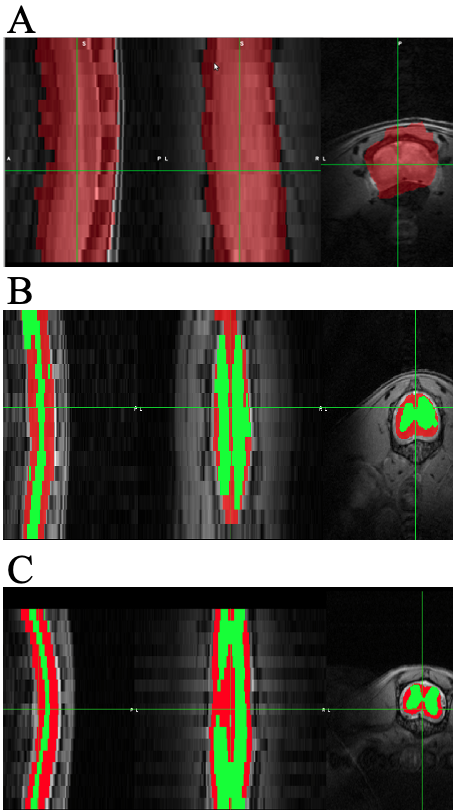


A

B

C

Figure S6. Retraining SCT for mouse spinal cord segmentation. (A) Before DL retraining, SCT failed to identify the whole cord. (B) After DL retraining using 8 subjects, SCT identified the whole cord but failed to segment the GM from the whole cord. (C) After DL retraining with additional 5 samples, SCT successfully segmented GM and WM.


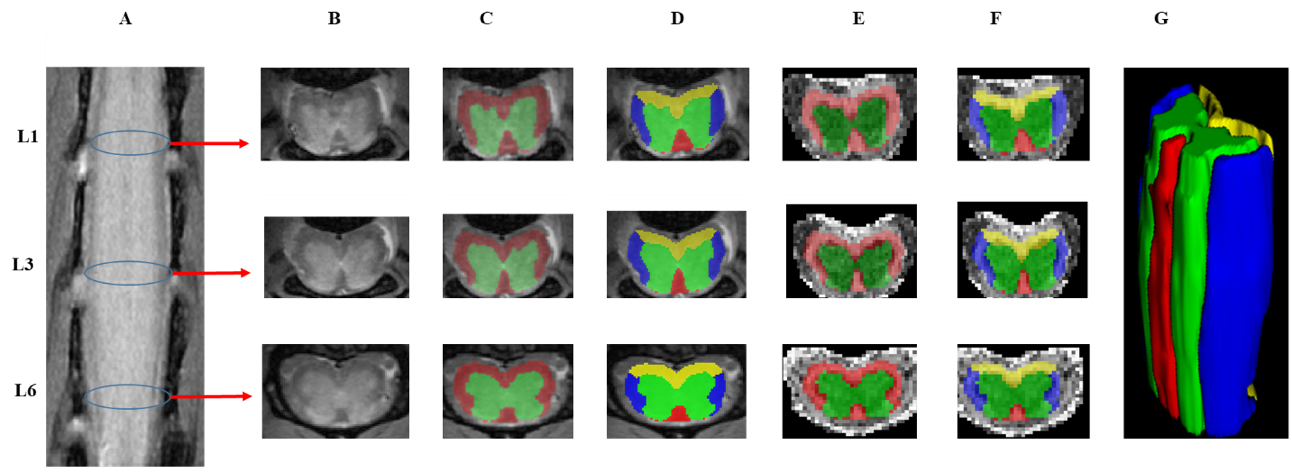


A

B

C

D

E

F

G

L1

L3

L6

Figure S7. Segmentation of the mouse lumbar spinal cord and the ROIs used for analysis. (A) Coronal view: three blue circles indicating the lumbar spinal cord at L1-L6. The largest lumbar enlargement is at L3. The area from L1 to L6 were covered by 9 slices. (B) High-resolution axial PD-W images showing distinct grey and white matter structures. (C) Grey matter (green) and white matter (red) segmentation using SCT. (D) Segmentation of WM into VF (yellow), LF (blue) and DF (red) ROIs. (E, F). Segmentation of FA maps using registered ROIs from C and D, respectively. (G) A 3-dimensional rendering of the 9 slices covering the area used for the lumbar region analysis.

**SUPPLEMENTARY MATERIAL 7: Daily EAE scores**

Figure S8. EAE progression. EAE scores were measured for healthy and EAE mice for 20 days. The error bars indicate standard deviation of the group clinical scores.

**SUPPLEMENTARY MATERIAL 8: MRS of the mouse brain**

| 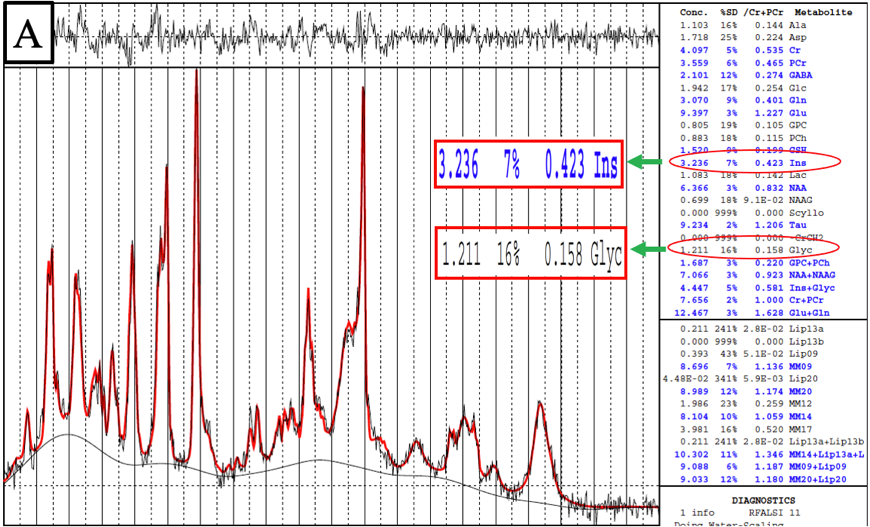 |
| --- |
| 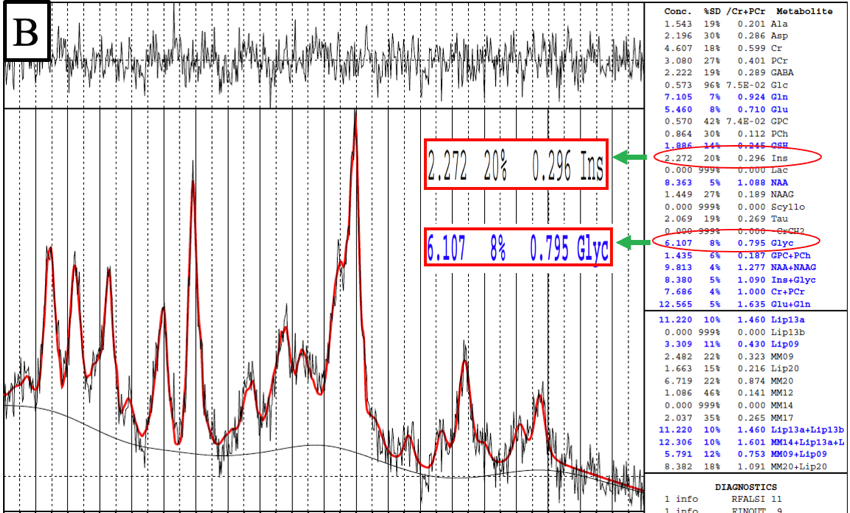 |

Figure S9. LCModel measurement of Gly and m-Ins in the brain and spinal cord. MRS were acquired with TE = 14 ms. (A) MRS of the brain and (B) of lumbar spinal cord. Green arrows highlighted that (i) Gly was detected in the lumbar spinal cord but not in the brain, (ii) m-Ins was detected in the brain but not in the lumbar spinal cord.

**SUPPLEMENTARY MATERIAL 9: Correlations between MRS, DTI, and EAE score**


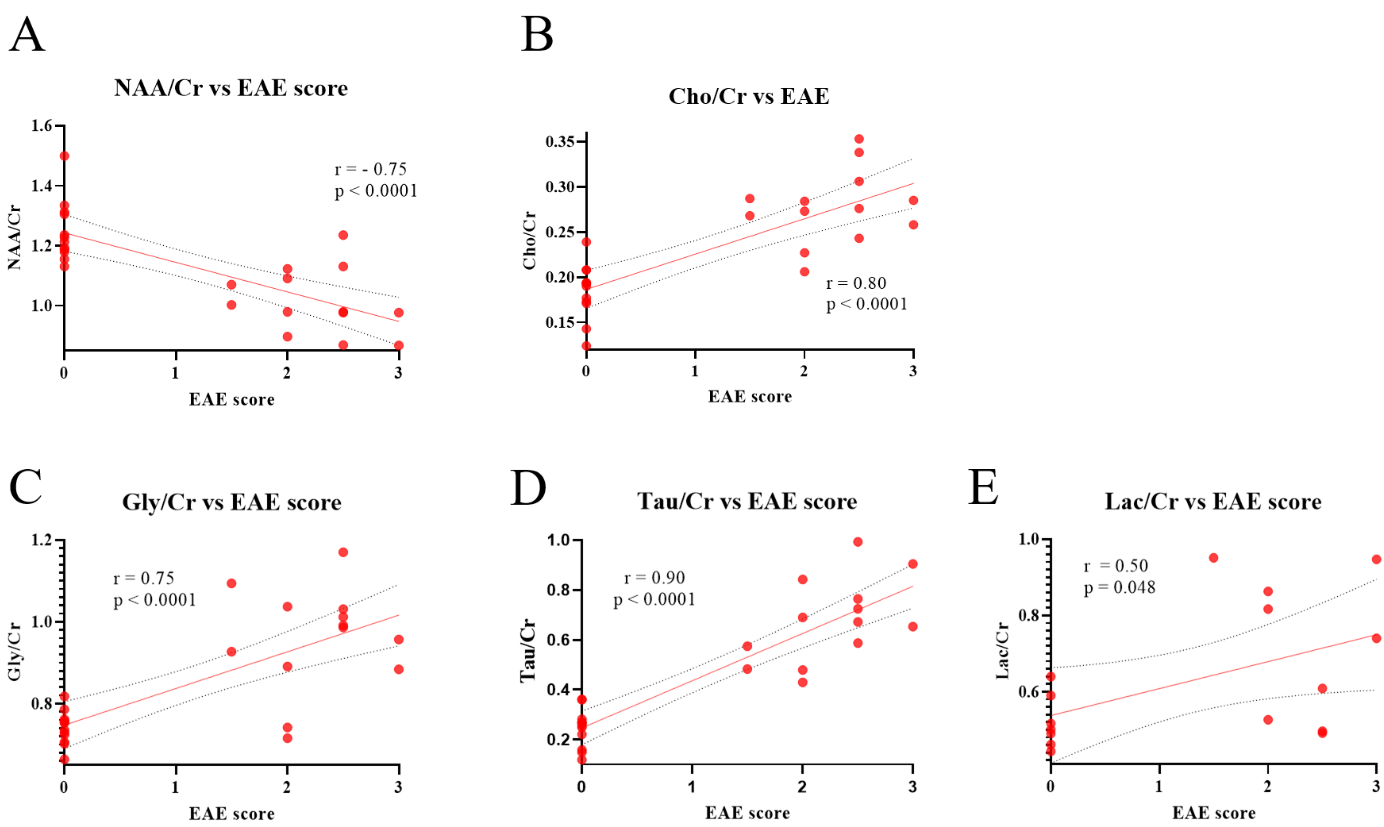


Figure S10. Correlation between EAE scores and MRS metabolites. (A) NAA/Cr, (B) Cho/Cr, (C) Gly/Cr, (D) Tau/Cr and (E) Lac/Cr.


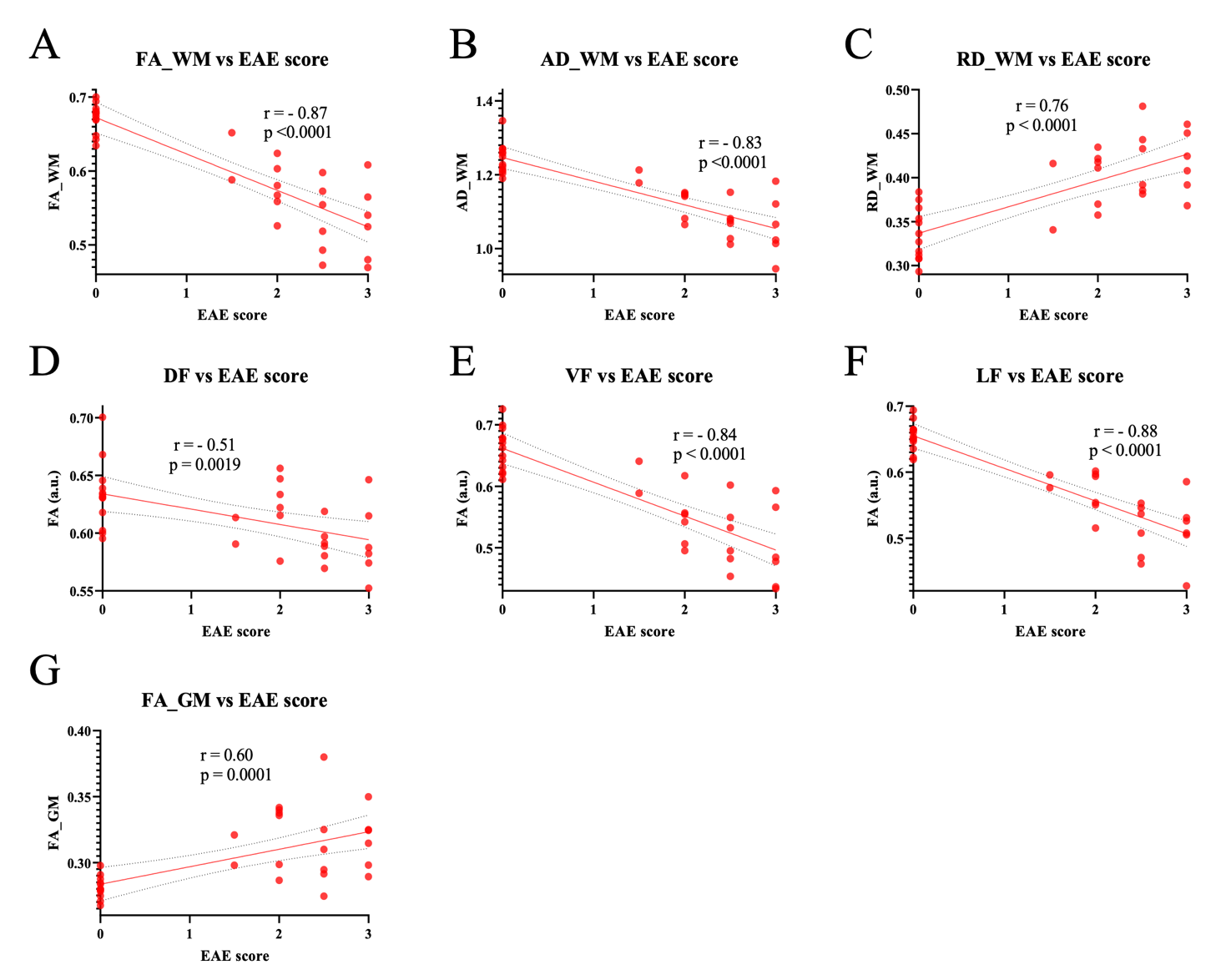


Figure S11. Correlations between EAE scores and DTI metrics. In the WM: (A) FA, (B) AD, (C) RD. Correlation of EAE scores with FA in three subregions of WM: (D) DF, (E) VF and (F) LF. In the GM: (G) FA.


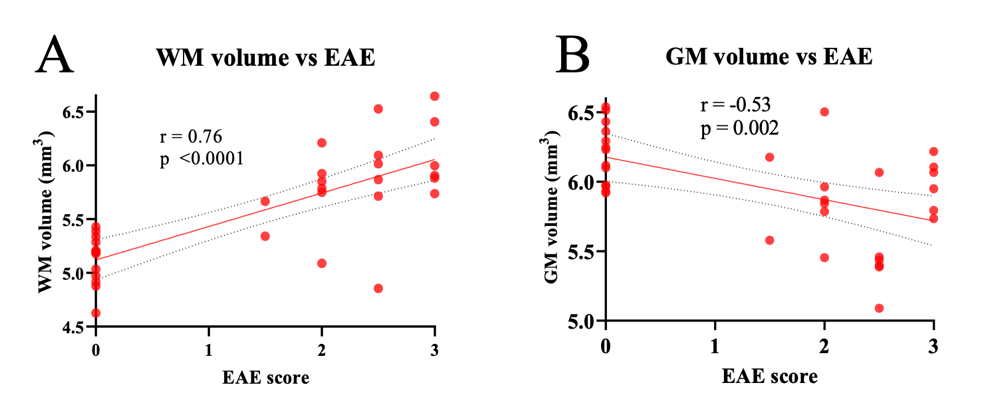


Figure S12. Correlations between EAE scores and the WM and GM volumes. (A) Positive correlation with the WM and (B) negative correlation with GM volumes.


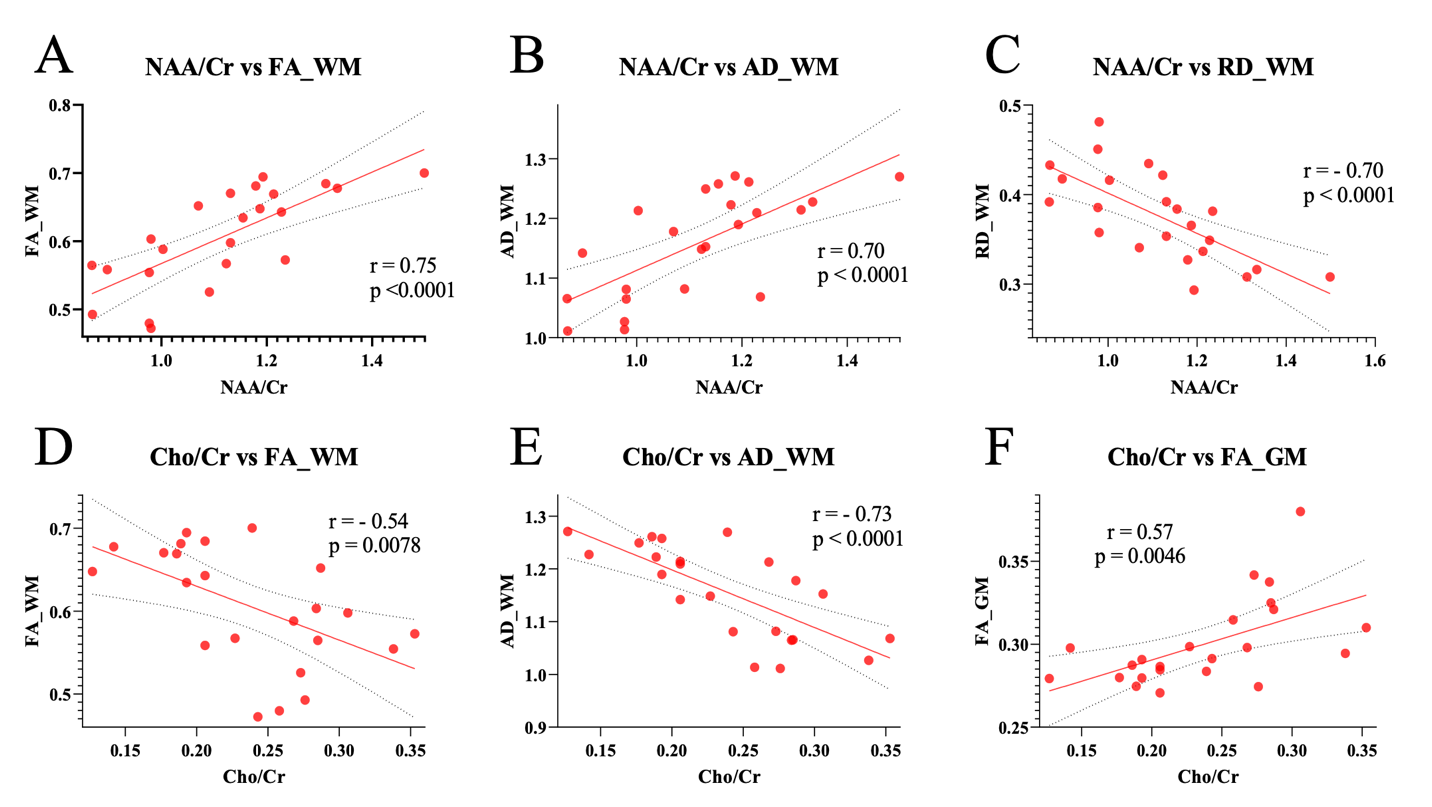


Figure S13. Correlations between MRS and DTI metrics. Strong correlations were observed between NAA and WM FA (A), AD (B) and RD (C). Cho had medium and strong correlations with WM FA (D) and WM AD (E), respectively, and medium correlation with GM FA (F).

**SUPPLEMENTARY MATERIAL 10: Phantom Gly + m-Ins**

**Validation experiments to resolve overlapping Gly and m-Ins peaks**

Gly and m-Ins are two of the metabolites of interest as biomarkers for EAE pathology. The assignment and quantification of Gly and m-Ins peaks, however, are problematic as the signals overlap with each other: Gly produces a singlet peak at 3.55 ppm, whereas m-Ins produces a multiplet of peaks between 3.45-3.65 ppm ^1,2^. Therefore, additional experiments were performed prior to data analysis to ensure validity of the measurements.

**MRS acquisition using Gly and m-Ins phantoms**

Three sets of phantoms were prepared: (*i*) Phantom I contained 200 mM m-Ins; (*ii*) Phantom II contained 66 mM Gly; (*iii*) Phantom III contained a mixture of 10 mM Gly and 10 mM m-Ins. Phantoms were made up in 20 mM sodium phosphate buffer at pH 7.4 and placed in 5 mL vials. Each of the phantoms were scanned using the same *in vivo* MRS protocol using various TE times. m-Ins has 6 protons and is modelled as an AM_2_N_2_P spin system. Protons A and P resonate at 4.05 ppm and 3.27 ppm respectively, whereas M_2_ resonates at 3.52 ppm and N_2_ resonates at 3.61 ppm.^3^ The J coupling constant between M and N is 9.99 Hz. With appropriate TE (ca. 30 ms), the spin-spin coupling results in J-evolution of M_2_ to an antiphase multiplet which allows for optimised Gly singlet detection (Figure S14 A).^4^

**Gly and m-Ins validation**

Phantom experiments

The *J*-evolution of m-Ins resulted in antiphase peaks at 3.65 ppm when the spectrum was acquired using TE = 30 ms compared to the in-phase peaks at TE = 14 ms (Figure S14 A). The Glycine peak did not show phase changes at these TEs because its peak is singlet (Figure S14 B). Consequently, when the phantom containing Gly/m-Ins mixture was scanned at TE = 30 there was a noticeable signal drop of m-Ins signal at 3.65 ppm and the Gly peak was less obscured (Figure S15).

| A  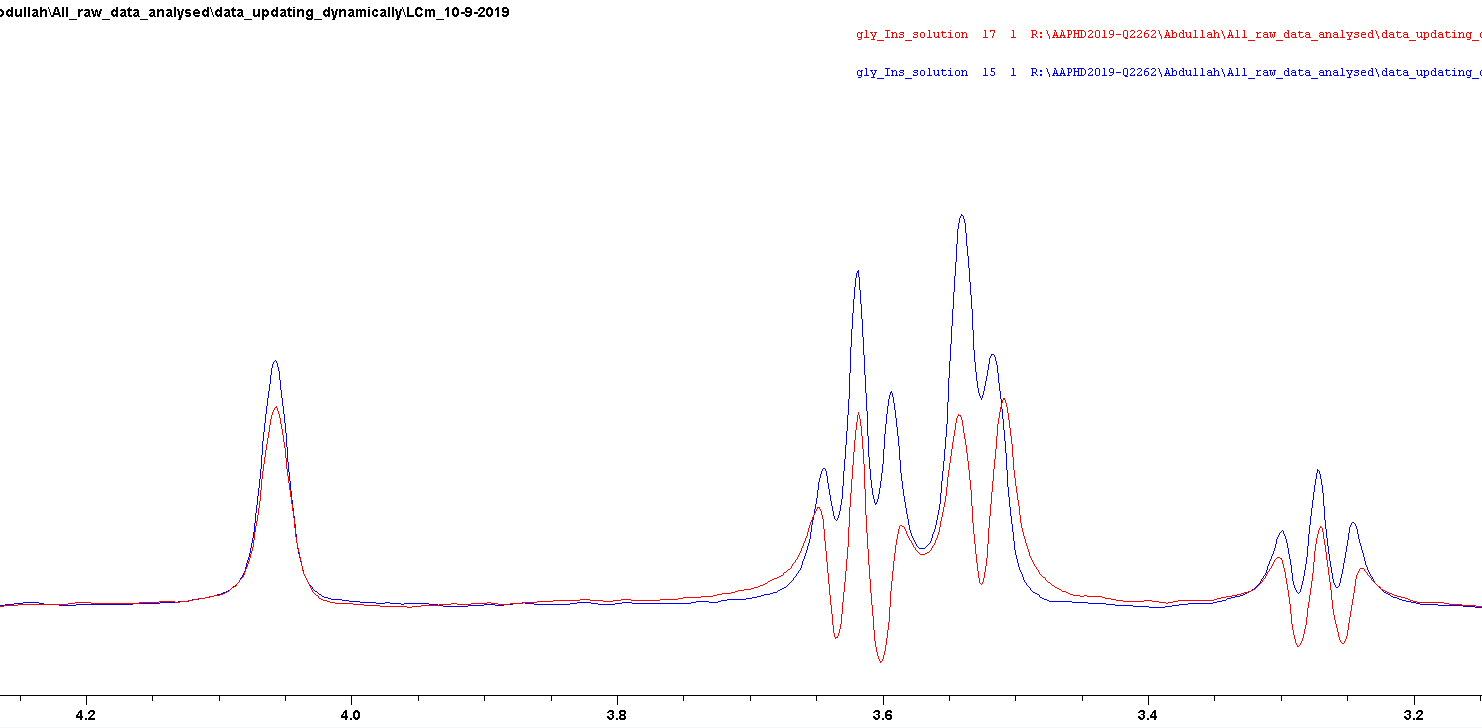 |
| --- |
| B  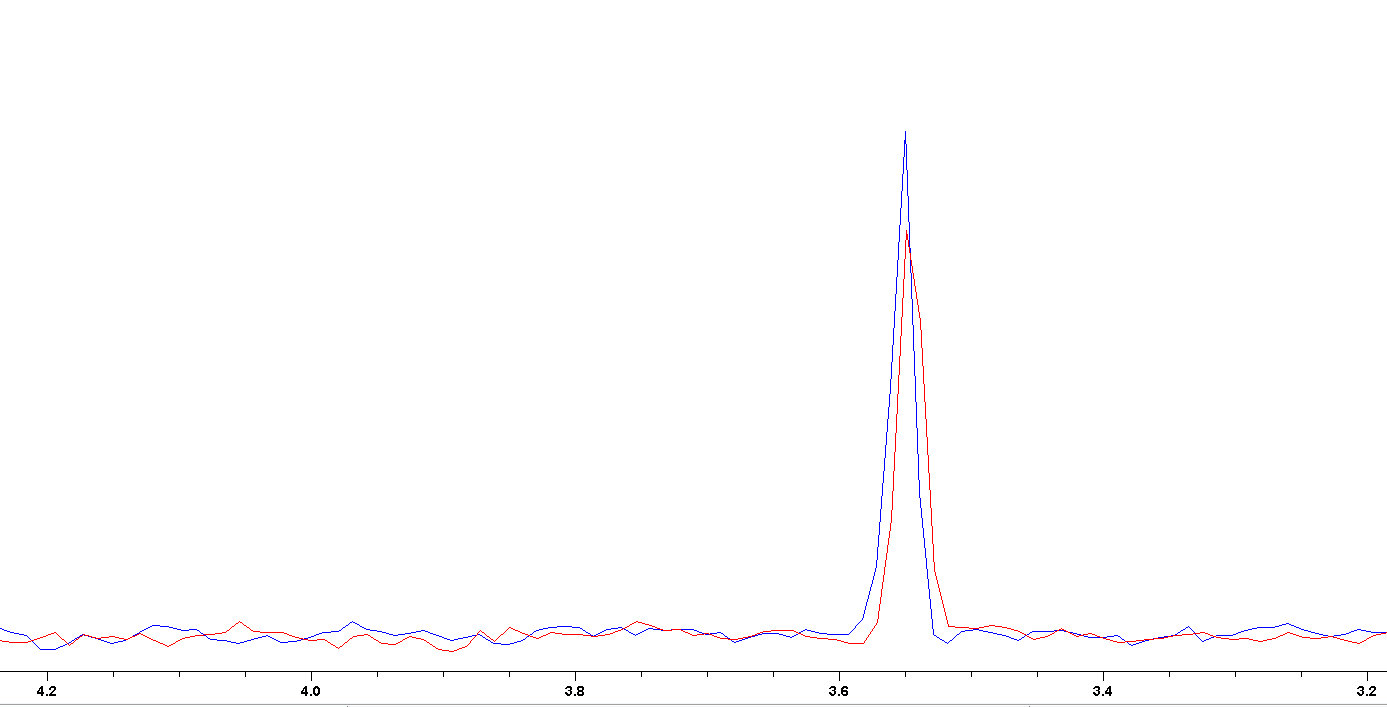 |

Figure S14. MRS of m-Ins (A) and Gly (B) acquired at TE 14 ms (blue) and TE 30 ms (red).


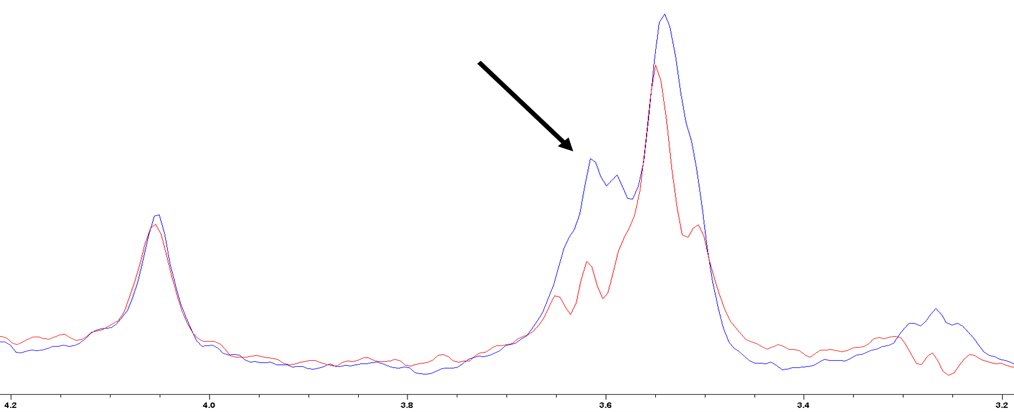


Figure S15. MRS of Gly/m-Ins mixture acquired at TE 14 ms (blue) and TE 30 ms (red). The arrow points to the signal drop of m-Ins at TE 30 ms at 3.65 ppm.

Below are absolute measurements (relative to water not to Cr) which did not change the overall conclusion.


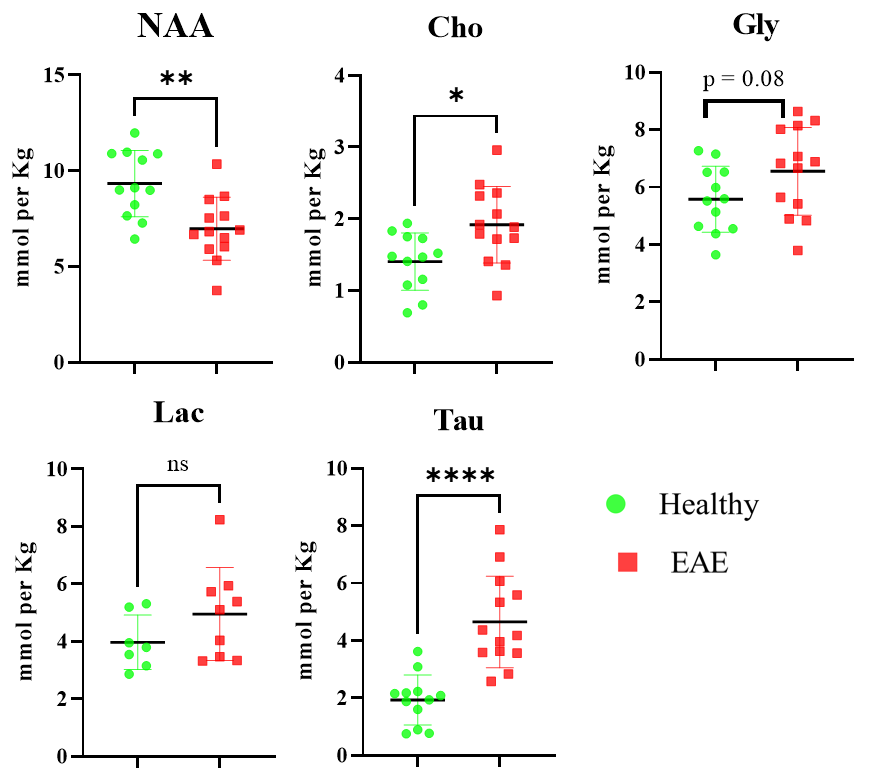


**Figure S16. Measurements of metabolites changes between healthy and EAE mice**.

**References for Supplementary Materials.**

1. Hattingen E, Lanfermann H, Quick J, Franz K, Zanella FE, Pilatus U. 1H MR spectroscopic imaging with short and long echo time to discriminate glycine in glial tumours. *MAGMA*. Feb 2009;22(1):33-41. doi:10.1007/s10334-008-0145-z

2. Patkee PA, Baburamani AA, Long KR, et al. Neurometabolite mapping highlights elevated myo-inositol profiles within the developing brain in down syndrome. *Neurobiol Dis*. Jun 2021;153:105316. doi:10.1016/j.nbd.2021.105316

3. Govindaraju V, Young K, Maudsley AA. Proton NMR chemical shifts and coupling constants for brain metabolites. *NMR Biomed*. May 2000;13(3):129-53. doi:10.1002/1099-1492(200005)13:3<129::aid-nbm619>3.0.co;2-v

4. Gambarota G, Xin L, Perazzolo C, Kohler I, Mlynárik V, Gruetter R. In vivo 1H NMR measurement of glycine in rat brain at 9.4 T at short echo time. *Magn Reson Med*. 2008;60(3):727-731.
